# Deciphering the Crosstalk Between Myeloid-Derived Suppressor Cells and Regulatory T Cells in Pancreatic Ductal Adenocarcinoma

**DOI:** 10.3389/fimmu.2019.03070

**Published:** 2020-01-22

**Authors:** Carole Siret, Aurélie Collignon, Françoise Silvy, Stéphane Robert, Thierry Cheyrol, Perrine André, Véronique Rigot, Juan Iovanna, Serge van de Pavert, Dominique Lombardo, Eric Mas, Anna Martirosyan

**Affiliations:** ^1^Aix Marseille Univ, CNRS, INSERM, CIML, Centre d'Immunologie de Marseille-Luminy, Marseille, France; ^2^Aix Marseille Univ, INSERM, CRO2, Centre de Recherche en Oncologie biologique et Oncopharmacologie, Marseille, France; ^3^Aix Marseille Univ, INSERM, VRCM, Centre de Recherche Vasculaire de Marseille, Marseille, France; ^4^Aix Marseille Univ, CEFOS, Centre d'exploration Fonctionnelle Scientifique, Marseille, France; ^5^Aix Marseille Univ, CNRS, INSERM, Institut Paoli-Calmettes, CRCM, Centre de Recherche en Cancérologie de Marseille, Marseille, France

**Keywords:** pancreatic cancer, immunosuppression, MDSC, Tregs, immune cell interactions

## Abstract

Pancreatic ductal adenocarcinoma (PDAC) is a fatal disease with rising incidence and a remarkable resistance to current therapies. The reasons for this therapeutic failure include the tumor's extensive infiltration by immunosuppressive cells such as myeloid-derived suppressor cells (MDSCs) and regulatory T cells (Tregs). By using light sheet fluorescent microscopy, we identified here direct interactions between these major immunoregulatory cells in PDAC. The *in vivo* depletion of MDSCs led to a significant reduction in Tregs in the pancreatic tumors. Through videomicroscopy and *ex vivo* functional assays we have shown that (i) MDSCs are able to induce Treg cells in a cell-cell dependent manner; (ii) Treg cells affect the survival and/or the proliferation of MDSCs. Furthermore, we have observed contacts between MDSCs and Treg cells at different stages of human cancer. Overall our findings suggest that interactions between MDSCs and Treg cells contribute to PDAC immunosuppressive environment.

## Introduction

Pancreatic ductal adenocarcinoma (PDAC) is a lethal malignancy projected to become the 2nd leading cause of cancer-related death in 2030 (1). With more than 337,000 new cases worldwide in 2012, it represents a major public health issue. The time-delayed diagnosis is due to the non-specific symptoms, as well as the lack of early detection markers (2). The median survival after diagnosis of PDAC is 4–6 months. The main reason of this poor prognosis is the resistance to most therapies, including current modalities of immune checkpoint blockade (1, 2). The therapeutic failure might result from the low level of immunogenicity of neoplastic cells, the tumor's robust immunosuppressive mechanisms, or both (2, 3). There is indeed a uniquely immunosuppressive tumor microenvironment (TME) dominant in most human PDAC. Increasing evidence suggests that the TME supports cancer initiation, progression and the development of metastasis (4, 5). The major drivers of the pro-tumorigenic microenvironment in PDAC include a highly fibrotic stroma and an extensive infiltration by immunosuppressive cell populations such as tumor-associated macrophages (TAMs), regulatory T cells (Tregs), and myeloid-derived suppressor cells (MDSCs). These tumor characteristics provide a barrier to the delivery of cytotoxic agents and limit effector T cell infiltration at the tumor site (4, 5). In addition, the presence of immunosuppressive cells hamper effector T cell recruitment and activation leading to a profound immune dysfunction (6). Thus, it is essential to understand the mechanisms of pancreatic cancer's immune evasion to translate effective immunotherapy in this disease. In this study, we have been particularly interested in MDSCs and Treg cells which represent an essential class of immunoregulatory cells in PDAC.

MDSCs are key regulators of immune responses in many pathophysiological conditions, including cancer (7, 8). MDSCs are a heterogeneous population of cells characterized by their myeloid origin and immature state (9). These cells are endowed with highly suppressive machinery and hamper both innate and adaptive immune responses *via* different mechanisms. For instance, MDSCs are able to inhibit effector T cells leading to the failure of efficient anti-tumor responses (7–9). In the context of PDAC, it has been shown that primary and metastatic PDAC cells secrete factors involved in the induction, recruitment and survival of myeloid cells leading to accumulation of MDSCs (10, 11). These cells are indeed expanded significantly in cancer patients and tumor-bearing animals in PDAC (12). Furthermore, the targeted depletion of an MDSC subset in mouse models of PDAC is shown to unmask the tumor to adaptive immunity (13). Last, but not least the accumulation of MDSCs in the peripheral circulation of patients has been related to the extent of disease, correlates with stage and is associated with a poor prognosis (14–16).

Treg cells are crucial in mediating immune homeostasis and promoting the establishment and maintenance of peripheral tolerance. These cells regulate a diverse array of immune responses in the context of autoimmunity, allergies, microbial infections, and cancers (17, 18). While generally beneficial in the former conditions, their inhibitory activity often antagonizes protective immunity in the latter settings (19). In the context of PDAC an increased Treg prevalence has been demonstrated to be a prognostic factor (12, 20). The recruitment of Tregs occurs early, as demonstrated by their presence in pre-malignant lesions, and their prevalence increases with pancreatic tumor progression (12, 20). Moreover, it has been shown that the depletion of Treg cells in PDAC slows tumor growth and prolongs survival (21, 22).

Recently a degree of crosstalk between these 2 major populations of suppressor cells has been suggested, but incompletely defined in different cancer models (23–26). A variety of mechanisms for these interactions have been proposed, including the ability of MDSCs to promote the *de novo* development/expansion/recruitment of Treg cells (23–26). Although a strong influx of MDSCs and Treg cells has been described in PDAC (12, 20–22), there is no evidence yet on the presence of interactions between these major immunoregulatory cells in pancreatic tumors. Moreover, many unresolved questions remain. For instance, whether Tregs act on MDSCs and shape their functional differentiation remains unclear. All in all, the mechanisms of immunosuppression in different cancers, including PDAC, have not been yet fully studied from the perspective of the interplay between these major immunoregulatory cell populations. In the current study, we have identified and characterized a crosstalk between MDSCs and Treg cells in murine and human PDAC tumors. Our results further revealed that the *in vivo* depletion of MDSCs led to a significant reduction of Treg cells in the pancreatic tumors. We have next investigated the cellular mechanisms of these interactions in PDAC. Our results show that (i) MDSCs are able to induce Treg cells in a cell-cell dependent manner, (ii) Treg cells affect the survival and/or the proliferation of MDSCs. Overall, the modulation of MDSC and Treg cell interactions to alleviate tumor-induced immunosuppression might suggest a new therapeutic solution for the PDAC.

## Materials and Methods

### Ethics Statement

The investigation was conducted in accordance with the French guidelines for animal care and the 2010/63/EU directive of the European Parliament, and was approved by the local ethics committee of Aix-Marseille University and by the Ministère de l'Enseignement Supérieur, de la Recherche et de l'Innovation. The protocols were registered under numbers APAFIS#4396-2016030709341791 and APAFIS#21966-2019091116114397. Mice were daily monitored for any behavioral and physical changes.

### Mice and Cell Lines

Eight to 10 week-old C57BL/6J Rj (H-2b) mice were purchased from Janvier (Le Genest-St. Isle, France). The syngeneic tumorigenic murine pancreatic carcinoma cell line Panc02 was cultured in RPMI 1640 10% FCS 100 units/mL penicillin (Invitrogen), 100 μg/mL streptomycin and were tested negative for mycoplasma contamination.

### Immunohistofluorescence

Frozen tissue sections were subjected to immunodetection after saturation with PBS 4% BSA. Sections were incubated with primary antibodies diluted in PBS 1% BSA. Gr-1^+^ and Foxp3^+^ and TCRγδ^+^ cells were detected by incubation with the rat anti-Gr-1 (BD Pharmingen), the rabbit anti-Foxp3 (Abcam), and the armenian hamster anti-TCRγδ (Biolegend) antibodies, respectively. After three washes in PBS, samples were incubated for 1 h with either Alexa Fluor 488-, Alexa Fluor 594- (life technologies) or Cy3-conjugated goat (Jackson Immunoresearch) immunoglobulin [Ig], raised against rat, rabbit and armenian hamster Igs, respectively, then washed in PBS. Nuclei were labeled with Draq5 and sections were mounted in ProLong Gold (Invitrogen). Confocal microscopy acquisitions were performed using a Leica SP5 microscope coupled with a Leica scanning device (Leica Microsystems, Mannheim, Germany). Images were recorded with LAS AF Lite acquisition software and were analyzed with the publicdomain ImageJ software (NIH; http://rsb.info.nih.gov/nih-image/).

### Immunohistochemistry

#### Mouse Tissues

Mouse pancreatic tumors were harvested, frozen in liquid nitrogen and cut into 8 μm thick sections. Frozen slides were incubated 20 min at room temperature (RT), fixed with cold acetone for 10 min at 4°C, air-dried at RT and proceed to staining. Briefly, slides were rehydrated in TBS and endogenous peroxidase activity was blocked with Bloxall solution (Vector laboratories) for 20 min at RT. Primary antibodies (CD4, CD8, Gr-1, and Foxp3 purchased from Abcam) were incubated for 2 h at RT. After washes in TBS, slides were incubated with avidin/biotin/peroxidase complex (Vectastain kit from Vector Laboratories). Antigen detection was performed by incubation with substrate-chromogen 3,3-diaminobenzidine (DAB, Vector Laboratories). Sections were counterstained with Mayer' hematoxylin and mounted with Faramount Mounting Medium, Aqueous (Agilent). Images were captured using a BH-2 Olympus microscope with X20 objective.

#### Human Tissues

Some tumor samples (*n* = 19) were obtained after pancreatic resection (duodeno-pancreatectomy) from patients diagnosed with PDAC (Gastroenterology and Digestive Surgery departments, Timone Hospital, Marseille, France; CRO2 Agreement DC20131857) between February 2007 and February 2016. All specimens were evaluated by an expert pathologist. Additionally, a pancreas adenocarcinoma tissue array (#PA484; 24 cases) was purchased from Pantomics (Euromedex, France) to complete the collection. This TMA was composed of 3 normal pancreas tissues, 1 islet cells tumor and 20 pancreatic adenocarcinoma (5 stage 1; 11 stage 2; 2 stage 3; and 2 stage 4). All those samples were in duplicate. Overall we analyzed 43 human samples.

Sections (5 μm) of formalin-fixed paraffin embedded tissue were used. After paraffin removal and antigen retrieval, pancreatic tissue sections were incubated with an anti-CD4, anti-CD8, anti-CD15, anti-CD11b, and anti-Foxp3 for 2 h at RT. After washing, slides were proceed as described in the section Mouse tissues. For double staining, we used the Polink DS-MR-Hu C1 Kit (GBI Labs) to detect the anti-Foxp3 in GBI-Permanent Red (Red) and the anti-CD11b in Emerald (Green).

### Orthotopic Tumor Induction

Subconfluent cultures of Panc02 cells were harvested using a 10% trypsin solution, washed twice in PBS and resuspended as single-cell suspension in matrigel. The pancreas of anesthetized mice was exposed after laparotomy. Panc02 cells were injected directly into the pancreas (0.8 × 10^6^ cell/100 μl matrigel) using a tuberculin syringe. After suturing, mice received sub-cutaneous injections of analgesic (Buprenorphine 0.1 mg/kg) after the operation and again a few hours later.

### Cell Suspension Preparation

Tumors were harvested 3 weeks after tumor cell inoculation and dissected into fragments with scissor followed by incubation with collagenase Type I-A from clostridium histolyticum (1 mg/ml in RPMI-2% FCS, Sigma Aldrich) for 30 min at 37°C under agitation. Cell suspensions were mixed every 10 min during agitation. The suspension was filtered through 70 μm strainer to remove macroscopic debris. Red blood cells were lysed using ACK Lysis buffer (Invitrogen) and cell suspension was filtered through a 30 μM strainer before flow cytometry staining and cell isolation.

### Antibodies and Flow Cytometry

The following antibodies were used for flow cytometry analysis: CD45-A700, CD3-A488, CD25-APC/eFluor780, LY6C-A488, CD11b-PE/Cy5, Ly-6G(Gr-1)-APC, CD69-APC/eFluor780, CD62L-PE/Cy5, LAP(TGF-β1)-PE/Cy7, CD115-APC/eFluor780, CD127-APC/eFluor780, CCR5-PE, and isotype controls (Rat IgG1 K Isotype Control PE/Cy7, Rat IgG2b K Isotype Control PE, Rat IgG2a K Isotype Control eFluor450, Rat IgG2a K Isotype Control PE) were purchased from eBioscience; Ly-6G-PE, CD4-PE/CF594, CD8a-V450 from BD Biosciences, CD124-PE/Cy7, CD40-PB, B7H1-BV421, CD103-APC/Cy7, CTLA4-BV421, CD45-PB, F4/80-PE/Dazzle™ 594 from Ozyme.

For intracellular staining of cytokines, cells were incubated for 4 h at 37°C with monensin (GolgiStop, BD Pharmingen) before the staining. Isolated cells from the spleens or tumors were incubated with anti-CD16/CD32 antibody (BD Pharmingen) to prevent non-specific antibody binding. Surface antigens were stained with the antibodies diluted in PBS 5% FCS 2 mM ETDA and incubated for 20 min at 4°C. Dead cells were excluded using LIVE/DEAD Fixable Aqua Dead Cell Stain (Invitrogen). Intracellular stainings were performed using the Foxp3/Transcription Factor Staining Buffer Set (Ebioscience). Annexin V (Ozyme) and 7AAD (Beckman Coulter) stainings was performed according to the manufacturer's recommendations. Multiparameter analysis were performed on a Gallios flow cytometer (Beckman Coulter) and analyzed with FlowJo software (Tree Star). All flow cytometric analysis of immune cells was performed on live CD45^+^ cells after excluding doublets.

### Immune Cells Isolation

MDSCs were purified either from the spleen or the tumor using the Myeloid-Derived Suppressor Cell Isolation mouse Kit (Miltenyi Biotec) according to the manufacturer's protocol. CD4^+^CD25^+^ and CD4^+^CD25^−^ T cells were magnetically enriched from either the spleen or the tumor using CD4^+^CD25^+^ Regulatory T Cell Isolation Kit, mouse (Miltenyi Biotec). CD4^+^ T cells were harvested after the first step of CD4^+^CD25^+^ cell's isolation. CD8^+^ T cells were isolated from the spleen of naive mice with EasySep™ Mouse Naïve CD8^+^ T Cell Isolation Kit (StemCell) according to manufacturer's instructions.

### T Cell Proliferation Assay

Responder cells (CD8^+^ or CD4^+^CD25^−^ T cells) were labeled with 2.5 μM CFSE (5 × 10^6^ cell/ml RPMI; 10 min at 37°C under agitation). CFSE labeled cells were then plated onto round bottom 96-well plates coated with CD3 antibodies (8 μg/ml; BD biosciences) in culture medium RPMI- PS-10% heat inactivated FCS, 50 μM β-Mercaptoethanol, 1% non-essential amino acids, and 1% sodium pyruvate. Purified suppressor cells (MDSCs or CD4^+^CD25^+^) were added in indicated ratios and plates were incubated at 37°C. The proliferation was measured by assessing dilution of CFSE by flow cytometry after 48 h (CD8^+^) or 72 h (CD4^+^CD25^−^) with CD3/CD28 stimulation (CD28 1 μg/ml). Controls were wells with responder cells without suppressor cells. Treg suppression assay were performed in the presence of IL-2 (50 U/ml).

### Detection of ROS and Arginase-1 Activity

The cell permeant reagent 2′,7′-dichlorofluorescin diacetate (DCFDA; Thermo Fisher Scientific) was used for the measurement of ROS production by MDSCs. 1 × 10^5^ purified MDSCs or CD8^+^ cells were incubated at 37°C in PBS without serum in the presence of DCFDA (2 μM) for 10 min, washed twice with cold PBS, before flow cytometry staining and analysis. Since the non-labeled cells cultured alone showed some auto-fluorescence, the ratio between the mean DCFDA fluorescence and the mean of cells auto-fluorescence was calculated.

For measuring arginase 1 activity, 2 × 10^5^ purified MDSCs were lysed on ice in 50 μl lysis buffer (0.1% Triton X100, 100 μg/ml pepstatin, 100 μg/ml aprotinin, and 100 μg/ml antipain). Arginase 1 activity was assessed in supernatant of frozen cell lysates using QuantichromTM Arginase assay kit (cat# DARG-100, BioAssays Systems) according to manufacturer' instructions.

### Videomicroscopy Acquisitions

A Lab-Tek chambered coverglass was coated with FCS. Purified CD4^+^CD25^+^ cells are loaded by 20 μM of green cell tracker during 30 min at 37°C. These cells are deposit with purified tumoral MDSCs with a 1/2 ratio in a same lab-tek well. Cells were then placed in a temperature- and CO_2_-controlled chamber mounted on an Olympus IX83 inverted microscope and incubated for 10 h. Images were captured every 20 min using an orca-flash4 camera [Hamamatsu] with a ×40 objective. All interactions persistent more than for 40 min, as well as transient interactions for 3 wells were quantified.

### Light Sheet Microscopy

#### Immunofluorescence Tumor Wholemount Stainings

Tumors were dissected and fixed overnight in 0.4% PFA/PBS at 4°C. Prior antibody staining, tumors were permeabilized (0.4% Triton X100, 1% milk/PBS) and subsequently blocked in block solution (0.4% Triton X100, 1% milk, 5% serum/PBS). Wholemount stainings were performed by using anti-alpha smooth muscle actin directly coupled with Alexa488, anti-Foxp3, and anti-Gr-1 as primary antibodies and Alexa-dye coupled secondary antibodies diluted in block solution. Following each staining step, samples were washed several times in PBS-Tx (0.42% Triton X100/PBS) and in PBS.

#### Optical Clearing and Wholemount Acquisition

Tumors were cleared before acquisition on the La Vision Ultramicroscope II (LaVision BioTec, Bielefeld, Germany). After dehydration in methanol (20, 40, 60, 80, 100%, each step 1 h and overnight in 100%, samples were optically cleared first in methanol/BABB (ratio 1:1) (benzyl alcohol:benzyl benzoate, ratio 1:2) 8 h and finally in BABB overnight. Stacks were captured with a step size of 4 μm and magnification 1X. 3D reconstruction, cell counting and analysis of cell interactions are performed by using IMARIS software (Version 9.1.0, Bitplane). The Matlab function associated with Imaris software quantified the number of cells in interaction. Since lymphocytes are 8–10 μm in diameter and myeloid cells are ~20–30 μm in diameter, here we considered that MDSCs and Treg cells are in interaction if the distance between their centroids is ≤ 20 μm.

### T Cell/MDSC Coculture

#### CD4^+^ T Cell/MDSC Coculture

MDSCs isolated from tumor were cocultured with CD4^+^ T cells purified from TB mice spleen at ratio (MDSCs:CD4^+^ T cells) 3:1 for 4 days in culture medium with CD3/CD28 stimulation. Co-cultures were performed in conventional dishes (96 wells U-bottom plate) or using *Transwell* chamber (0.4 μm pore) to separate the 2 cell populations; MDSCs and CD4^+^ T cells were added in the upper and lower chambers, respectively. The percentage of CD4^+^Foxp3^+^ cells was evaluated by flow cytometry.

#### CD4^+^CD25^+^ Treg Cell/MDSC Coculture

MDSCs isolated from tumor were cocultured with CD4^+^CD25^+^ cells purified from spleen or tumor onto round bottom 96-well plates in culture medium with CD3/CD28 stimulation at indicated ratios. The viability of MDSCs was analyzed by flow cytometry after 24 and 48 h of co-culture and the percentage of CD4^+^Foxp3^+^ cells was evaluated after 4 days.

### MDSC Depletion

To deplete MDSCs *in vivo*, mice received 2 *i.p*. injections of RB6-8C5 antibody (anti-Gr-1, 200 μg/mice diluted in sterile PBS, BioXCell) 11 and 14 days after tumor cells inoculation. Control mice received PBS or isotype control antibody (clone LTF-2, BioXCell). Animals were euthanized 3 days after the second injection, spleens and tumors were harvested to determine the efficiency of Ab-mediated depletion. For comparisons between two groups, statistical analyses were performed using a Student's *t*-test and *p* < 0.05 was considered significant.

## Results

### PDAC Is Characterized by a Strong Accumulation of Immunosuppressive Cell Populations

**Figure 1:**
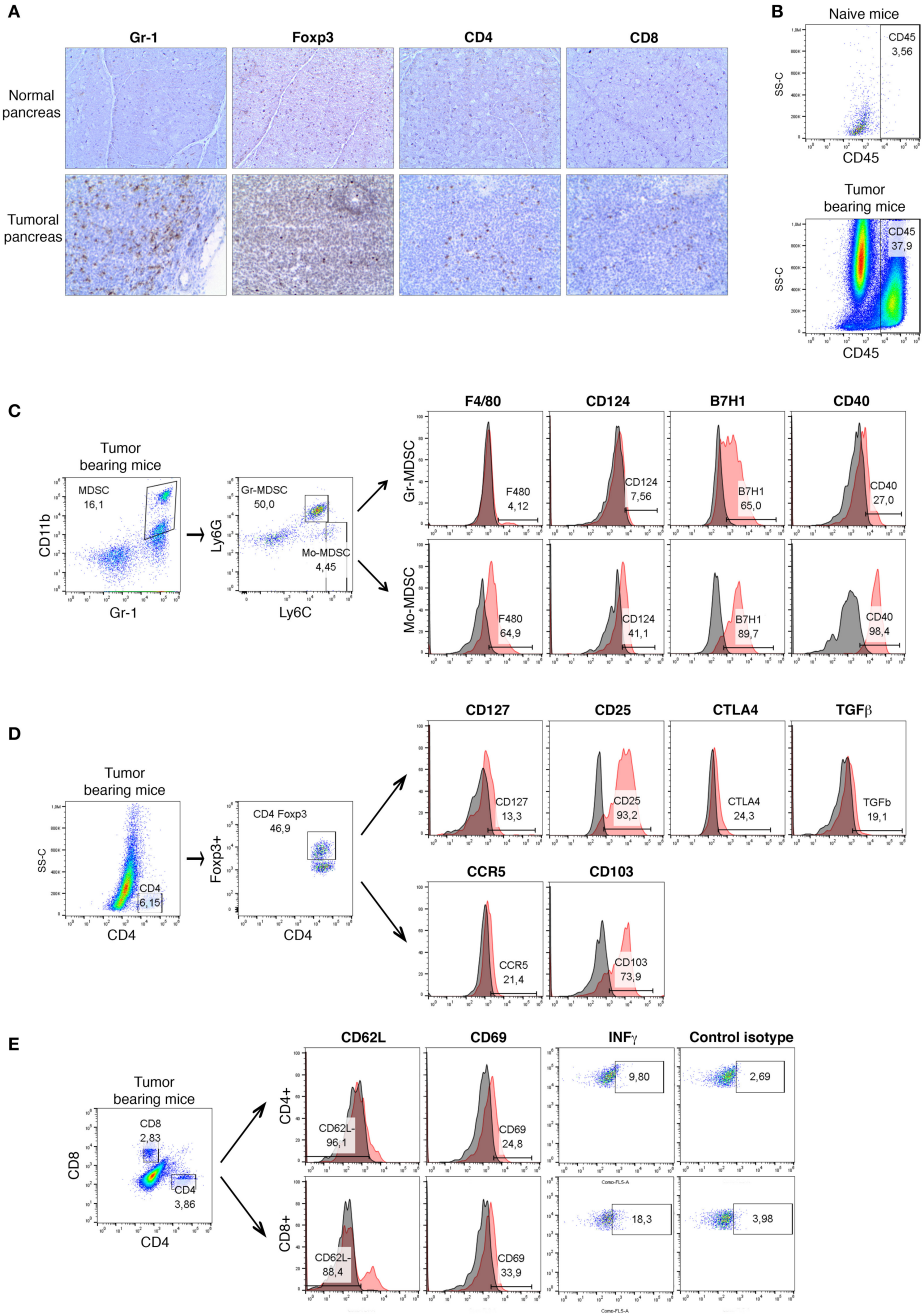
A detailed phenotypical analysis of the immune cell populations in orthotopic mouse model of pancreatic cancer. Panc02 cells were injected in the pancreas of C57BL/6 mice (*n* = 5) and tumors were harvested 3 weeks post- inoculation. **(A)** Representative IHC analysis of Gr-1 (granulocytes), CD4, CD8, and Foxp3 (regulatory T cells) on normal pancreas (*n* = 3) and on pancreatic tumor (*n* = 5) frozen sections. Original magnification x100. Flow cytometry analysis **(B)** of CD45^+^ immune cells in normal pancreas and pancreatic tumors, **(C)** of surface molecules F4/80, CD124, CD40, and B7H1 on Gr-MDSC (CD11b^+^Gr-1^+^Ly6G^+^Ly6C^low^) and Mo-MDSC (CD11b^+^Gr-1^+^Ly6G^−^Ly6C^high^) in pancreatic tumors, **(D)** of surface molecules CD25, CD127, CCR5, CTLA4, TGF-β, and CD103 on Treg cells (CD45^+^CD4^+^Foxp3^+^) in tumors, **(E)** of activation markers CD62L and CD69, as well as the intracellular expression of IFN-γ on CD4^+^ and CD8^+^ T cells in tumors. Gray histograms represent isotype control and red histograms specific staining as indicated. Percentage of positive cells are shown. Three independent experiments have been performed with similar results. Representative dot plots and histograms for one of these experiments are shown. See also Supplementary Figures 1, 2.

An orthotopic mouse model of pancreatic cancer (27), which mimics human PDAC with regard to histological appearance, and the pattern of the disease, was used in this study. By using immunohistochemistry and multiparametric flow cytometry methods, we have performed a detailed phenotypical analysis of the immune cell populations following tumor induction (Figure 1). A strong influx of immune cells characterized by an extensive infiltration of MDSCs and Treg cells in the pancreas (Figure 1) and the spleen (Supplementary Figure 1) of tumor-bearing (TB) mice was observed.

MDSCs consist of two major subsets based on their phenotypic and morphological features: granulocytic Gr-MDSCs (Ly6G^+^Ly6C^low^) and monocytic Mo-MDSCs (Ly6G^−^Ly6C^high^). At first, we examined the expression of surface molecules either associated with MDSCs (F4/80 and CD124) or involved in MDSCs-mediated immunosuppression (B7H1 and CD40) on CD11b^+^Gr-1^+^ cells in tumors (Figure 1C) and spleens (Supplementary Figure 1). We could observe a strong expression of B7H1 and CD40 on both MDSC subsets derived from tumors. However, the upregulation of F4/80 and CD124 was more substantial on Mo-MDSCs (Figure 1C). In order to study the systemic immune response following tumor induction, we analyzed the phenotype of these MDSC subsets. Splenic Mo-MDSCs derived from TB mice upregulated F4/80, CD124, and CD40 compared to Gr-MDSCs (Supplementary Figure 1B). Both subsets showed a strong expression of B7H1 at their cell surface (Supplementary Figure 1B).

As shown in the Figure 1D and Supplementary Figure 2, the expression of Treg-associated molecules and functional markers was then analyzed. The transcriptional factor Foxp3 serves as a lineage specification factor of murine Treg cells. Currently the most commonly used markers for Treg identification and characterization are CD4, Foxp3, CD25 (IL-2R alpha), and CD127 (IL-7R alpha). Figure 1D demonstrates that most of tumoral CD4^+^Foxp3^+^ Treg cells were CD25^high^CD127^low^. Tregs manifest their immunosuppressive function through the secretion of immunosuppressive soluble factors such as TGF-β, as well as cell contact mediated regulation *via* molecules like CTLA-4 (17, 18). Our data shows that Treg cells derived from tumors have an increased expression of CTLA4 and TGF-β at their surface as compared with splenic counterparts (Figure 1D and Supplementary Figure 2A). We have then studied the amounts of CD103 (Integrin α_E_) that has been reported to be a hallmark of tumor-infiltrating regulatory T cells in colon cancer (28). Recent studies have described CD103^+^ Tregs as potent suppressors of anti-tumor immune responses in TB mice (28). In our model of murine PDAC, 70% of tumoral Treg cells expressed high levels of CD103 (Figure 1D). It has been shown that chemokine receptors CCR5 could mediate trafficking of Treg to PDAC tumors (21). Cells within the TME, such as pancreatic stellate cells and tumor-infiltrating MDSCs, have also been reported to express high levels of Treg cell chemotactic factors, including CCL5 (20). We examined the expression of these chemokine receptors on CD103^+^ Tregs in the tumors and spleen of PDAC mice. As show in Figure 1D, 20% of tumoral Treg cells up-regulated CCR5 at their surface as compared with splenic counterparts. Moreover, we have observed that at least 60% of CD4^+^Foxp3^+^CCR5^+^ cells exhibited high surface amounts of CD103, while at least 30% of those cells upregulated CTLA4 (data not shown). In contrast, the expression levels of CTLA4, CD103 and CCR5 on splenic Treg cells from TB mice (Supplementary Figure 2A) were reduced as compared with tumor infiltrating Treg cells (Figure 1D).

In parallel, the recruitment and the activation of CD4^+^ T and CD8^+^ T cells was analyzed (Figure 1E). A low infiltration of both cell populations was observed in the tumors of PDAC mice. To determine whether the intratumoral T lymphocytes show evidence of activation and thus potentially contributed to efficient antitumor immunity, we further assessed the surface marker expression (CD62L and CD69) and the production of IFN-γ (Figure 1E). The latter is an essential cytokine in anti-tumoral immunity. While the expression of CD62L is rapidly lost upon activation, the CD69 molecule is induced on activated T cells. More than 88% of intratumoral CD4^+^ T and CD8^+^ T cells from PDAC mice strongly down-regulated CD62L (Figure 1E). Moreover, the expression of CD69 at the surface of 24% of CD4^+^ lymphocytes and 33% of CD8^+^ T cells was detected. Since phenotypic markers cannot conclusively determine if infiltrating T cells are truly naive or functionally inactivated, we have then measured the intracellular levels of effector molecule IFN-γ. Our data show that 10% of CD4^+^ T and 18% of CD8^+^ T cells from tumor produce IFN-γ suggesting an activated phenotype (Figure 1E). In contrast, splenic CD4^+^ T and CD8^+^ T cells showed low levels of CD69 expression (Supplementary Figure 2B). Furthermore, the production of IFN-γ by tumoral CD4^+^ T cells was higher as compared to their splenic counterparts (Figure 1E and Supplementary Figure 2B).

Overall, our data indicate a strong influx of immune cells (CD45^+^) following tumor induction in the pancreas of PDAC mice. We could observe an extensive myeloid cell (CD11b^+^Gr-1^+^) infiltration, as well as a strong recruitment of functionally active Treg cells into the pancreas of tumor-bearing mice. Furthermore, we identified different phenotypes of Treg cells for the expression of functional molecules depending whether the local or systemic immune responses were studied. Small numbers of activated effector T cells were present in the PDAC mice.

Thus, consistent with previous data in the genetically engineered mouse model of pancreatic cancer (KPC mice) (12), we have found that PDAC is characterized by a strong accumulation of immunosuppressive cell populations (MDSCs, Treg cells) associated with low levels of activated CD4^+^ and CD8^+^ T cells.

### MDSCs and Treg Cells From Pancreatic Tumors Are Able to Suppress Effector Responses

**Figure 2:**
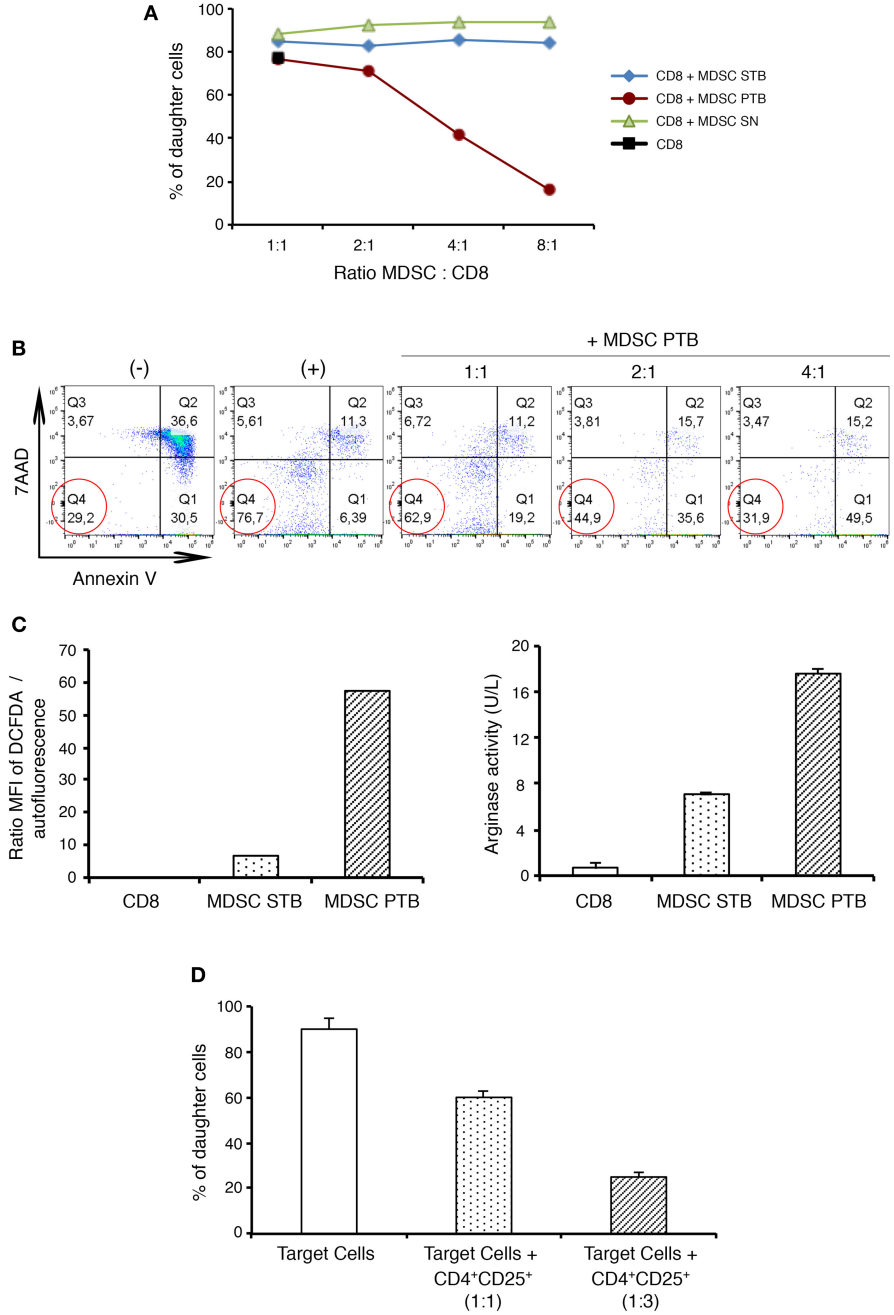
Functional characterization of MDSCs and Treg cells in PDAC. CD8^+^ T cells proliferation is suppressed by tumoral MDSCs. **(A)** CFSE labeled CD8^+^ T cells were cultured with MDSCs isolated either from naive spleen (MDSC SN) or from tumor-bearing mice spleen (MDSC STB) or from tumor (MDSC PTB) at different ratios. The percentage of CD8^+^ daughter cells was evaluated by flow cytometry after 48 h of culture with CD3/CD28 stimulation. **(B)** Tumoral MDSCs induce the death of CD8^+^ T cells *ex vivo*. CD8^+^ T cells were stimulated by CD3/CD28 and cultured with MDSCs isolated from tumor (MDSC PTB). Lymphocytes were then stained with cell death markers such as 7AAD and Annexin V. (–) no stimulation; (+) CD3/CD28 stimulation. **(C)** Production of ROS and arginase by MDSCs isolated either from the spleen (MDSC STB) or the tumor (MDSC PTB) of PDAC mice. CD8^+^ T cells were used as control. Cell lysates were analyzed for arginase 1 activity by measuring the ability to convert L-arginine to urea as describe in Materials and Methods. ROS production was determined by the production of fluorescent DCFDA as described in Materials and Methods. **(D)** Naive CD4^+^CD25^−^ T cells (Target cells) proliferation is suppressed by tumoral Tregs. CFSE labeled CD4^+^CD25^−^ T cells were cultured with Treg (CD4^+^CD25^+^) cells isolated from tumor. After 72 h of culture in presence of CD3/CD28 stimulation the percentage of CD4^+^ daughter cells was evaluated by flow cytometry.

MDSCs have been shown to enhance tumor growth by inhibiting immune responses and T cell proliferation (9). To determine the suppressive potential of this population, isolated CD11b^+^Gr-1^+^ cells either from the spleen of naïve mice (MDSC SN) or the spleen (MDSC STB) and pancreas (MDSC PTB) of tumor-bearing mice were incubated with CD8^+^ T cells in both proliferation and apoptosis assays. As shown in the Figure 2A, at a 2:1 myeloid-to-T cell ratio, MDSCs derived from tumors (MDSC PTB) slightly decreased T cell proliferation. Moreover, at the higher myeloid-to-T cell ratios observed *ex vivo* in mice with PDAC, tumoral MDSCs exhibited a strong ability to suppress T cell proliferation in a dose-dependent manner (Figure 2A). In contrast, CD11b^+^Gr-1^+^ cells isolated from the spleen of naïve or TB mice did not show any suppressive capacity (Figure 2A). Tumoral MDSCs also induced apoptosis of activated T cells (Figure 2B). Thus, MDSCs from pancreatic tumors can both suppress T cell proliferation and promote T cell death.

We next examined the presence of MDSCs functional molecules (arginase-1, ROS) which are essential for the immunosuppressive capacity of these cells (Figure 2C). We could observe an important up-regulation of ROS by MDSCs from PDAC tumors in contrast to CD11b^+^Gr-1^+^ cells isolated either from the spleen of naïve or TB mice (Figure 2C). Moreover, the tumoral MDSCs showed 3-fold more arginase-1 activity than their splenic counterparts (Figure 2C).

We then investigated the suppressive function of the other major immunoregulatory population in PDAC, the Treg cells (Figure 2D). Isolated CD4^+^CD25^−^ splenic T cells from PDAC mice were used as responders after *in vitro* CD3/CD28 stimulation. We then added isolated autologous tumoral CD4^+^CD25^+^ T cells and found that they were able to suppress CD3-induced proliferation of responder cells in a dose-dependent manner (Figure 2D). In these suppression assays, we identified Treg as CD4^+^CD25^+^ cells rather than CD4^+^Foxp3^+^ cells, because staining cells for Foxp3, a transcription factor, requires permeabilization. Since CD25 is also expressed by activated T cells, we evaluated CD25 expression on non-Treg populations (i.e., CD4^+^Foxp3^−^ T cells) in the pancreas of TB mice. In contrast to the majority of CD4^+^Foxp3^+^ T cells from the tumoral pancreas that expressed CD25, <30% on average of CD4^+^Foxp3^−^ T cells were CD25^+^ in these mice (data not shown). These findings confirm the minimal activation status of effector T cells in these pancreatic neoplasms.

To summarize, our findings reveal that tumoral MDSCs display a strong suppressive capacity characterized by the inhibition of effector CD8^+^ T cells proliferation, the induction of CD8^+^ T cell death, as well as the production of functional molecules (arginase 1, ROS). Moreover, we have shown that Treg cells isolated from the tumors were able to suppress the proliferation of CD4^+^ T cells.

### Direct Interactions Between MDSCs and Treg Cells in PDAC

**Figure 3:**
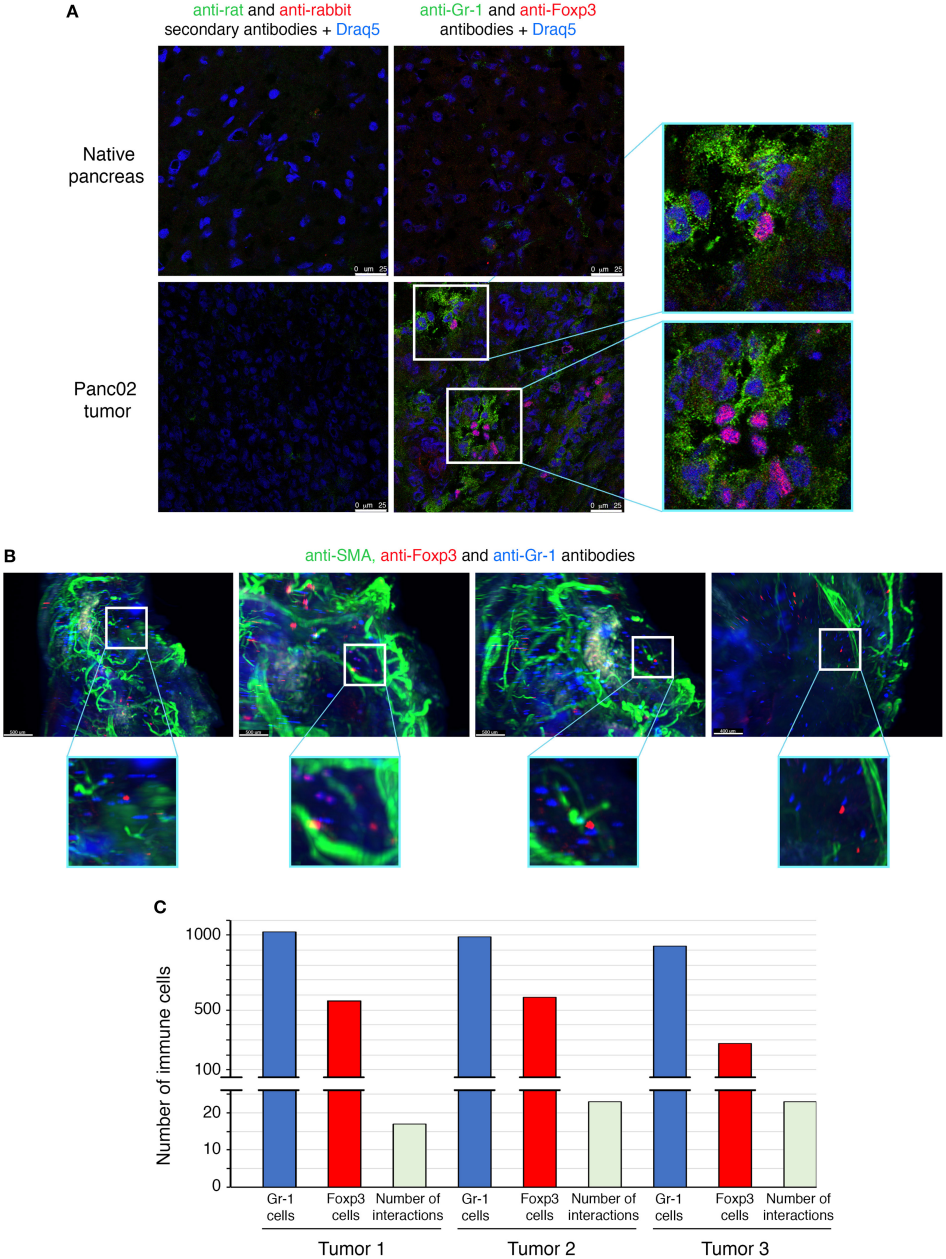
Direct interactions between MDSCs and Treg cells in orthotopic mouse model of pancreatic cancer. Panc02 cells were injected in the pancreas of C57BL/6 mice and tumors were harvested 3 weeks post-inoculation. **(A)** Representative IHF (confocal microscopy) analysis of Gr-1 (MDSCs in green) and Foxp3 (Treg cells in red) on normal pancreas and on pancreatic tumor frozen sections. The nuclei (in blue) are stained with Draq5, a fluorescent DNA marker. Zooms show proximity between the different immune cells. Scale bar 25 μm. **(B)** Light sheet microscopy analysis of Gr-1 (MDSCs in blue) and Foxp3 (Treg cells in red) on whole cleared pancreatic tumor. Zooms show closed contacts between the two populations. Blood vessels are stained with a pericyte marker, the alpha-smooth actin (in green). Scale bar 500 μm. **(C)** Histogram represents quantification of interactions whole cleared tumors calculated with a matLab function associated with the Imaris software. See also Supplementary Figure 3 and Supplementary Videos 1–3.

The interactions between Treg cells and MDSCs in different cancer models have been proposed to play a critical role in shaping the tumoral immunosuppressive environment. However, there is no evidence so far whether this crosstalk involves direct interactions between the two classes of immunoregulatory cells. To determine whether there are interactions between MDSCs and Treg cells in PDAC, we employed multicolor immunofluorescence imaging on tumor sections (Figure 3). We first determined the prevalence within the TME of cells expressing Foxp3, the lineage specification transcription factor of Treg cells, as well as of cells expressing Gr-1, defining the MDSC populations (Figure 3A). Gr-1^+^ and Foxp3^+^ cells were absent in the normal pancreas, but abundantly present within the neoplastic lesions of PDAC mice. The immune staining of tumor sections showed that some Foxp3^+^ cells are located in close proximity to cells expressing Gr-1 (Figure 3A).

To further study MDSCs/Tregs interplay, we have used light sheet fluorescent microscopy (LSM), which allows long-term live 3D imaging of organism models. In our study, we have used LSM for the 3D imaging of cell interactions in whole tumors which is technically challenging and has never been reported. Since only 4% of tumor-infiltrating CD45^+^ cells are positive for CD4 (Figure 1E) and only 50% of those are Treg cells (Figure 1D), the interactions between MDSCs and Treg cells are rare events to record and quantify. LSM technology applied to the whole pancreatic tumors allowed us to observe some of Foxp3^+^ Treg cells to directly contact Gr-1^+^ cells (Figure 3B and Supplementary Video 1). The quantification of these interactions has been performed on 3 different tumors (*ex vivo*) by using IMARIS software (Figure 3C). Consistent with the co-localization pattern observed on tumor sections (Figure 3A), we could observe more than 15 interactions between Treg cells and MDSCs in each analyzed PDAC tumor (Figure 3C). To highlight the real significance of MDSC and Treg interactions, we have to analyze their dynamic during tumor formation and progression. We have therefore quantified these interactions 1, 2, 3, and 4 weeks post-tumor induction. To do so, the tumors have been harvested at these different time points and both immunofluorescence and LSM imaging (*ex vivo* tumors) were performed. The immunofluorescence data (Supplementary Figure 4A) and LSM videos (Supplementary Videos 4–7) show an increase of MDSC and Treg cell interactions 2 weeks post-tumor induction (Supplementary Figures 4A,B, Supplementary Videos 4, 5). The numbers of co-localizing cell populations reach a peak 3 weeks after tumor inoculation (Supplementary Figures 4A,B, Supplementary Video 6). Interestingly, at later time points (4 weeks post-tumor inoculation) we have observed an accumulation of MDSC in clusters and outside of the core tumor, while Treg cells are still found inside the tumor (Supplementary Figures 4A, 5, Supplementary Video 7). There were fewer MDSC and Treg interactions at this stage of tumor progression. These qualitative LSM observations were quantified and confirmed as illustrated in the Supplementary Figure 4B. Moreover, no similar interactions were observed between MDSCs and γδT cells in the TME by using the same methodology (Supplementary Figure 6). Overall, our findings revealed a direct physical contact between MDSC and Treg cell in whole murine pancreatic tumors.

In order to evidence these physical interactions between Treg cells and MDSCs, we have performed videomicroscopic analysis (**Supplementary** Figure 3, Supplementary Videos 2, 3). Treg (CD4^+^CD25^+^) cells purified both from spleen and pancreas of tumor-bearing mice were incubated with tumoral MDSCs and the co-cultures monitored every 20 min (Supplementary Figure 3A). Moreover, the persistent or transient interactions were quantified (Supplementary Figure 3B). Our data demonstrates physical interactions between Treg (CD4^+^CD25^+^) cells and MDSCs in PDAC.

**Figure 4:**
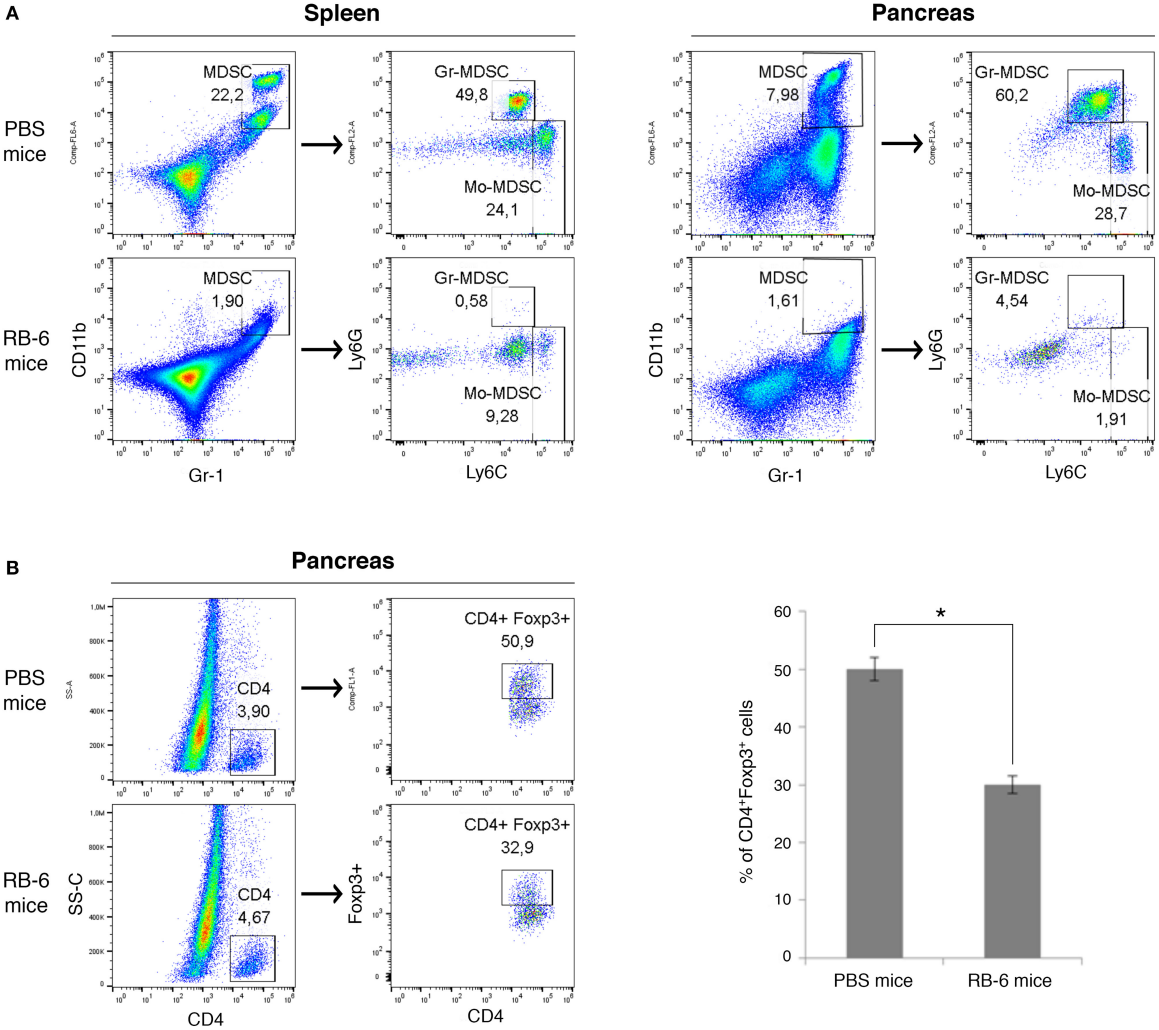
Systemic depletion of MDSCs inhibits the recruitment of Foxp3^+^ Tregs in pancreatic tumors. Mice were injected with Panc02 tumor cells on day 0 and Gr-1^+^ cells were depleted on day 15 and 18 with intraperitoneal injection of anti-RB6 antibody (RB-6 mice). Control mice received injection of PBS (PBS mice) or isotype control antibody. Spleens and tumors were harvested 3 weeks after PDAC induction. The percentage of splenic and pancreatic MDSCs (CD11b^+^Gr-1^+^), MDSC sub-populations **(A)** and Treg cells (CD4^+^Foxp3^+^) **(B)** was evaluated by flow cytometry. Percentage of positive cells are shown. The results represent one of three independent experiments with similar data, ^*^p < 0.05.

To evaluate the influence of MDSCs on accumulation of Tregs cells *in vivo*, we depleted Gr-1^+^ cells in PDAC mice (Figure 4). The injection of anti-Gr-1^+^ antibody resulted in near complete elimination of MDSCs in spleens and pancreas of PDAC mice as assessed by flow cytometry (Figure 4A). Tumor masses did not generally decrease in size during this treatment window (not shown). Remarkably, *in vivo* depletion of Gr-1^+^ cells in tumor-bearing mice led to a significant reduction in Treg cells (CD4^+^Foxp3^+^) in the pancreatic tumors (Figure 4B). In summary, our findings show that the systemic depletion of MDSCs inhibits the recruitment and/or induction of Foxp3^+^ Tregs in pancreatic tumors.

### A Cell-Cell Dependent Crosstalk Between Tumoral MDSCs and Treg Cells

**Figure 5:**
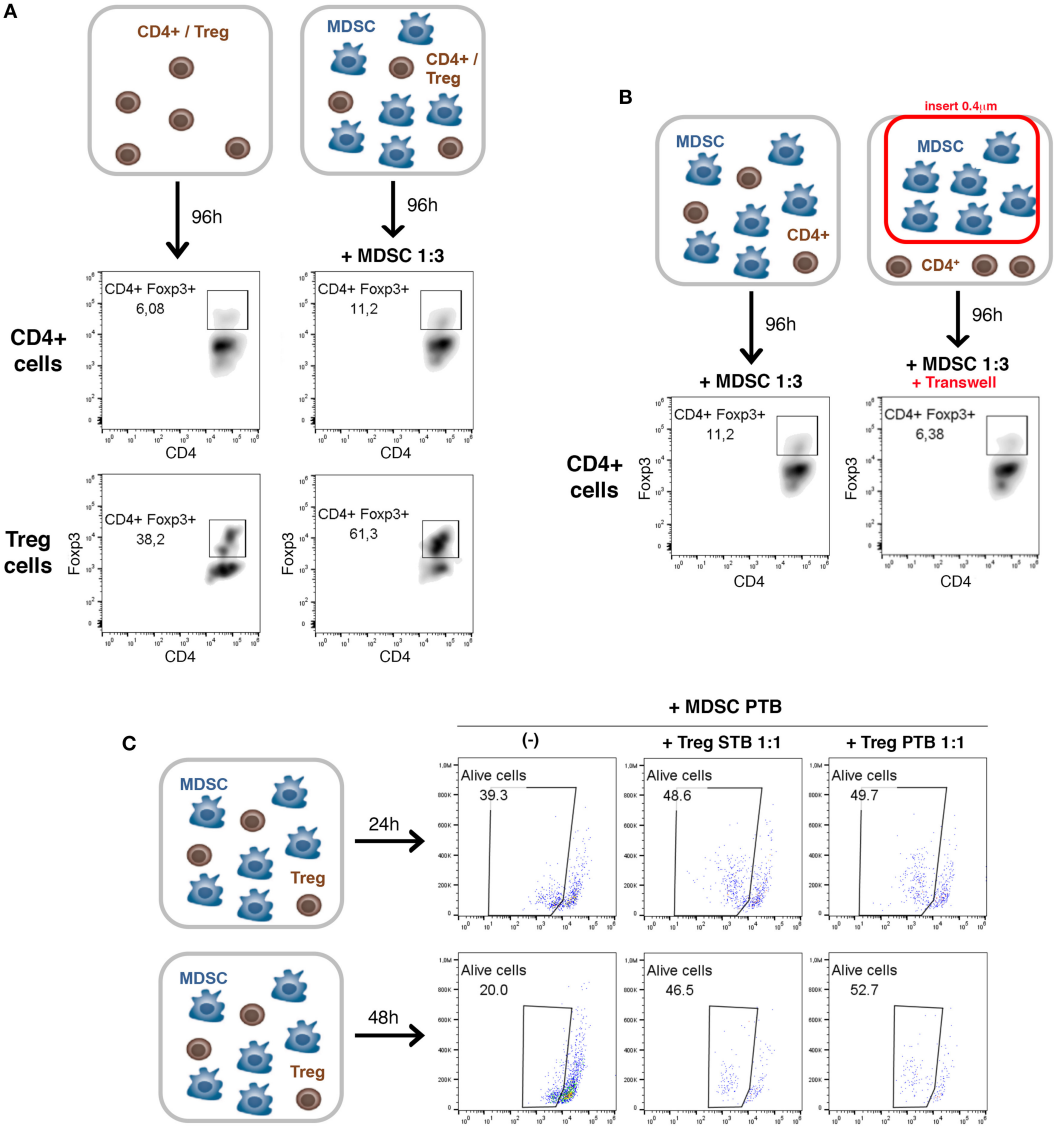
A cell-cell dependent crosstalk between tumoral MDSCs and Treg cells. **(A)** Purified CD4^+^ T cells or Treg cells (CD4^+^CD25^+^) from TB mice were cocultured with tumoral MDSCs. The percentage of CD4^+^Foxp3^+^ cells was evaluated by flow cytometry after 4 days of culture in the presence of CD3/CD28 stimulation. **(B)** Purified CD4^+^ T cells from TB mice were cocultured with tumoral MDSCs at 1:3 ratio in conventional dishes or using Transwell chamber to separate the 2 cell populations. The percentage of CD4^+^Foxp3^+^ cells was evaluated after 4 days by flow cytometry. **(C)** Purified Treg cells (CD4^+^CD25^+^) from spleen of tumor-bearing mice (Treg STB) or tumor (Treg PTB) were cocultured with tumoral MDSCs for 24 or 48 h. The viability of MDSCs was then assessed by flow cytometry using *LIVE*/*DEAD* Fixable *Aqua* Dead stain.

We further investigated the cellular mechanisms of MDSC and Treg interactions in PDAC (Figure 5). Several studies have described the ability of MDSCs to promote the *de novo* development/expansion/recruitment of Treg cells in different tumor settings (23–26). To determine whether MDSCs in PDAC possess this ability, *ex vivo* co-culture assays were carried out (Figures 5A,B). We incubated either purified CD4^+^ T cells or Treg cells with tumoral MDSCs at 1:3 ratio (Figure 5A). After 4 days of co-culture, we could observe in both conditions tested an increase of Treg cells in presence of tumoral MDSCs (Figure 5A). These data are consistent with our *in vivo* observations of MDSCs-dependent Treg cell recruitment and/or induction in PDAC (Figure 4). To identify whether soluble molecules or cell-cell interaction are involved in this process, we incubated purified CD4^+^ T cells and MDSCs in conventional dishes or using *Transwell* system (0.4 μM) to separate these 2 immune cell populations (Figure 5B). The induction of Treg cells by MDSCs was lost in the Transwell system suggesting that MDSC-mediated development/expansion of Treg cells was due to cell-to-cell interactions (Figure 5B).

Little is known on the converse impact of Treg cells on MDSCs. A novel strategy was described during the development of murine melanomas whereby Tregs shape the functional differentiation of MDSCs through the B7H1 pathway (23). Furthermore, it has been shown in B16 melanoma model that the expansion, recruitment, and activation of MDSCs occur in a Treg-dependent manner (29). In order to determine the impact of Treg cells on MDSCs in PDAC, we have performed *ex vivo* assays (Figure 5C). Purified Treg cells (CD4^+^CD25^+^) from spleen of tumor-bearing mice (Treg STB) or tumor (Treg PTB) were co-cultured with tumoral MDSCs. After 24 and 48 h of co-culture the percentage of alive MDSCs strongly increases in the presence of Treg cells isolated from TB mice (Figure 5C). Our findings reveal that Treg cells affect the survival and/or the proliferation of tumoral MDSCs.

Our results show that (i) MDSCs are able to induce Treg cell proliferation and/or development in a cell-cell dependent manner, (ii) Treg cells affect the survival and/or the proliferation of MDSCs.

### Identification of MDSC and Treg Cell Crosstalk in Human PDAC

**Figure 6:**
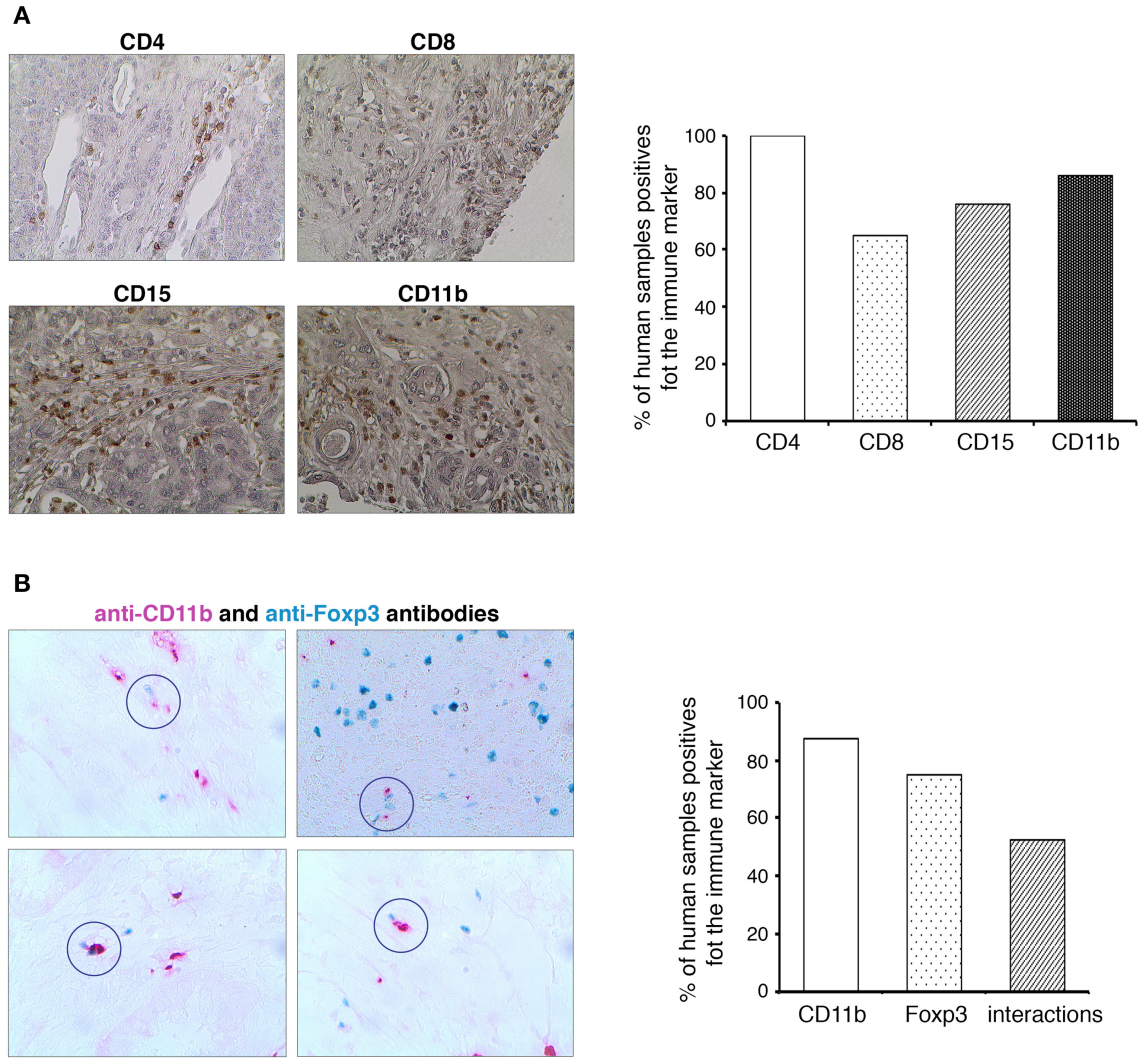
Identification of MDSCs and Treg cells crosstalk in human PDAC. **(A)** Formalin-fixed, paraffin-embedded tissue sections of human pancreatic cancer were stained with anti-CD4, anti-CD8, anti-CD15, and anti-CD11b antibodies. All immunohistochemistry experiments were performed with the Vector Kit. Histograms show the quantification of the different immune markers expression in PDAC. Original magnification x100. **(B)** Formalin-fixed, paraffin-embedded tissue sections of human pancreatic cancer were stained with anti-Foxp3 (in Emerald) and anti-CD11b (in Permanent-red) antibodies. All immunohistochemistry experiments were performed with the GBI Labs Kit. A visual evaluation of interactions between Foxp3^+^ and CD11b^+^ cells was performed on human PDA tissues. Histograms show the quantification of the markers expression in PDAC and the presence of their interactions.

To determine whether the MDSC and Treg cell interactions are present also in the human pancreatic cancer, we have studied the expression of different immune markers (CD4, CD8, CD15, CD11b, Foxp3) in a cohort of PDAC patients (Figure 6). Similar to mouse, there are also two types of human MDSCs. Both types express CD11b; however, there is no equivalent to the mouse Gr-1 marker. Instead, human M-MDSCs are characterized by their expression of CD14 and PMN-MDSCs by their expression of CD15 (8, 9). We could not observe any immune cell infiltration in normal human pancreas (not shown). As shown in the Figure 6A, almost 100% of the human PDAC tissues were infiltrated by CD4^+^ T cells, CD8^+^ T cells, and CD15^+^ myeloid cells. Moreover, CD11b^+^ cells were observed in 60% of studied samples. Since the Foxp3 staining was weak, we have used GBI Labs Kit to amplify the signal. We could observe the presence of Foxp3^+^ cells in more than 70% of tested samples (Figure 6B). By using the same experimental approach, we have identified and quantified the proximity between human CD11b^+^ and Foxp3^+^ cells. In more than 50% of analyzed PDAC patients, we have observed contacts between these cells (Figure 6B). Taken together these results suggest that MDSC and Treg interplay could also be present in human PDAC.

## Discussion

A complex relationship between the immune system and the development of pancreatic cancer has been largely described in patients and animal models. The progress in basic and translational immunology has confirmed the importance of these interactions in PDAC's prevention and prognosis (30). Neoplastic cells activate tumor-specific immune responses, but simultaneously trigger a strong immunosuppression, which is considered to be one of the main reasons for current immune-based therapy's failure (2, 5). Among the hallmarks of the immune dysfunction observed in PDAC is the recruitment and activation of immunosuppressive cells such as myeloid-derived suppressor cells and regulatory T cells. These latter shield neoplastic cells from immune detection and inhibit anti-tumoral effector responses (31).

Since the interactions between MDSCs and Treg cells are proposed to be a powerful barrier against anti-tumoral immunity in different cancer models (23–26), we have been particularly interested to study this interplay in the context of PDAC. Consistent with previous data in the genetically engineered mouse model of pancreatic cancer (KPC mice) (12), we have found that PDAC is characterized by a strong accumulation of immunosuppressive cell populations (MDSCs, Treg cells) associated with low levels of activated effector T cells. By using the innovative approach of light sheet fluorescent microscopy in whole pancreatic tumors, we have identified for the first time in a tumoral context the interactions between MDSCs and Treg cells which occurs *via* a direct physical contact. Our results further revealed that this interplay had a biological relevance since the *in vivo* depletion of MDSCs led to a significant reduction of Treg cells in the pancreatic tumors. Our findings on the biological relevance of these interactions are in concert with a previous study showing that the combination of *Listeria* vaccination and Treg depletion in a mouse model of PDAC shapes the functional differentiation of MDSCs.

Another new aspect of our study was MDSC/Treg interplay's contribution to the establishment and/or development of the immunosuppressive environment in PDAC. To better understand the mechanisms of PDAC-infiltrating MDSC and Treg crosstalk, we have used a Transwell system which allows to separate the two major immunoregulatory cell populations. Our results show that tumoral MDSCs are able to induce Treg cell proliferation and/or development in a cell-cell dependent manner. Moreover, we could observe that Treg cells affect the survival and/or the proliferation of MDSCs in PDAC. At present the molecular partners involved in the MDSC/Treg interplay are not fully understood and further studies will be needed to investigate these pathways. One candidate to consider could be the B7H1 pathway (23). It has been proposed during the development of murine melanomas whereby Tregs shape the functional differentiation of MDSCs through the B7 family molecules (23). Moreover, investigating the role of CD40 and CD80 molecules in the MDSCs/Treg crosstalk in PDAC could be promising. Indeed, the presence of these co-stimulatory molecules on MDSCs in the mouse colon and ovarian carcinomas models, respectively, is associated with Treg cells accumulation and/or functions (32, 33). Although inquiring the role of these candidates in the interactions between MDSCs and Treg cells in PDAC should be elucidated, it is important to highlight that they have an ectopic expression on different immune cell populations. This is a major obstacle for a future targeting of the crosstalk *via* their blocking by inhibitory antibodies for example. A broad transcriptomic approach might be a better tool to identify new and specific molecules involved in MDSC/Treg cell interplay in PDAC. In addition, the latter has also been reported in lung, colorectal and breast cancers, as well as in melanoma and B-cell lymphoma, suggesting that a contact-based crosstalk between these cell populations may be a general feature of tumor immune evasion (23–26). Moreover, based on our data and several other indications of a concerted immunosuppressive activity of Tregs and MDSCs in different cancer models, we expect the interactions between these major immunoregulatory cells to play a key role in PDAC development and progression.

The ability to effectively engage cancer immunity in therapies is highly promising and very challenging. The limitations encountered thus far in applying immunomodulatory strategies such as αCTLA4 and αPD1/PDL1 in PDAC to stimulate an endogenous T cell response may be the result of the profoundly suppressive effects of MDSCs and Treg cells (30, 31). Moreover, it is becoming increasingly clear that to improve the effects of conventional immune strategies in PDAC it may be necessary to target multiple forms of immune suppression simultaneously. On the other hand, safety becomes a counterbalancing concern, lest autoimmunity and organ dysfunction ensue. In summary, our study provides insights into MDSC/Treg cell crosstalk in PDAC which may help to explain the highly immunosuppressive nature of the pancreatic tumors. Should the interactions between MDSCs and Treg cells impact the tumoral progression, it will be highly promising to target/modulate this interplay to reverse tumor-induced immunosuppression and provide an efficient therapeutic strategy for the treatment of PDAC.

## Data Availability Statement

All datasets generated for this study are included in the article/Supplementary Material.

## Ethics Statement

The animal study was reviewed and approved by the local ethics committee of Aix-Marseille University and the Ministère de l'Enseignement Supérieur, de la Recherche et de l'Innovation. approved protocol numbers: APAFIS#4396 and APAFIS#21966.

## Author Contributions

AC, CS, SR, FS, VR, SP, DL, EM, and AM performed the *in vitro* and *in vivo* experiments. TC and PA helped with the *in vivo* experiments. AM conceived and supervised the study. AC, CS, SR, VR, EM, and AM analyzed data. CS, DL, VR, JI, EM, and AM contributed to discussion and wrote, illustrated, reviewed, and edited the manuscript. DL, JI, EM, and AM have acquired fundings. All authors approved the submitted version of the manuscript.

### Conflict of Interest

The authors declare that the research was conducted in the absence of any commercial or financial relationships that could be construed as a potential conflict of interest.

## Supplementary Material

Supplementary Figure 1 Characterization of MDSCs, Treg cells and effector T cells in the spleen of PDAC mice. C57BL/6 mice were injected with Panc02 cells in the pancreas and spleens were harvested 3 weeks post-inoculation. Spleens from naive mice were used as controls. Flow cytometry analysis of **(A)** MDSC recruitment and **(B)** of the expression of surface molecules F4/80, CD124, CD40, and B7H1 on Gr-MDSCs (CD11b^+^Gr-1^+^Ly6G^+^Ly6C^low^) and Mo-MDSCs (CD11b^+^Gr-1^+^Ly6G^−^Ly6C^high^) was performed. Gray histograms represent isotype control and red histograms specific staining as indicated. Percentage of positive cells are shown. Three independent experiments have been performed with similar results. Representative dot plots and histograms for one of these experiments are shown.

Supplementary Figure 2 Characterization of Treg cells and effector T cells in the spleen of PDAC mice. C57BL/6 mice were injected with Panc02 cells in the pancreas and spleens were harvested 3 weeks post-inoculation. Spleens from naive mice were used as controls. Flow cytometry analysis of **(A)** the surface molecules CD127, CCR5, CTLA4, TGF-β, and CD103 on Treg (CD45^+^CD4^+^ Foxp3^+^), **(B)** the activation markers CD62L and CD69, as well as the intracellular expression of IFN-γ on CD4^+^ and CD8^+^ T cells. Gray histograms represent isotype control and red histograms specific staining as indicated. Percentage of positive cells are shown. Three independent experiments have been performed. Representative dot plots and histograms for one are shown.

Supplementary Figure 3 Assessment of MDSC and Treg interactions in pancreatic tumors by videomicoscopy. Treg (CD4^+^CD25^+^) cells purified both from spleen or pancreas of tumor-bearing mice were loaded with FITC cell tracker and incubated with tumoral MDSCs for 10 h. Microscopic acquisition was performed every 20 min **(A)** and the percentage of total CD4^+^CD25^+^ cells bound to MDSCs was quantified 5 and 10 h post-co-culture. The ratio of persistent interactions (more than 40 min) and transient interactions was also reported **(B)**. ^***^Mann and Whitney analysis with *p* value < 0.001%.

Supplementary Figure 4 Dynamic interactions between MDSCs and Treg cells in orthotopic mouse model of pancreatic cancer. Panc02 cells were injected in the pancreas of C57BL/6 mice and tumors were harvested 1, 2, 3, and 4 weeks post-inoculation (three tumors per week). **(A)** Representative IHF (confocal microscopy) analysis of Gr-1 (MDSCs in green) and Foxp3 (Treg cells in red) on pancreatic tumor frozen sections for each week. The nuclei (in blue) are stained with Draq5, a fluorescent DNA marker. Zooms show proximity between the different immune cells. Scale bar 25 μm. **(B)** Light sheet microscopy analysis of Gr-1 (MDSCs) and Foxp3 (Treg cells) on whole cleared pancreatic tumor. Histogram represents quantification of interactions from three whole cleared tumors calculated with a matLab function associated with the Imaris software. See also Supplementary Video 4 (1 week post-tumor inoculation), Supplementary Video 5 (2 weeks post-tumor inoculation), Supplementary Video 6 (3 weeks post-tumor inoculation), and Supplementary Video 7 (4 weeks post-tumor inoculation).

Supplementary Figure 5 Direct interactions between MDSCs and Treg cells in the center and boundary of tumors harvested at 3 and 4 weeks post-inoculation (three tumors per week). Representative IHF (confocal microscopy) analysis of Gr-1 (MDSCs in green) and Foxp3 (Treg cells in red) on pancreatic tumor frozen sections for each week. The nuclei (in blue) are stained with Draq5. Zooms show proximity between the different immune cells. Scale bar 50 μm. See also Supplementary Video 6 (3 weeks post-tumor inoculation) and Supplementary Video 7 (4 weeks post-tumor inoculation).

Supplementary Figure 6 Assessment of interactions between MDSCs and γδT cells in orthotopic mouse model of pancreatic cancer. Panc02 cells were injected in the pancreas of C57BL/6 mice and tumors were harvested 1, 2, 3, and 4 weeks post-inoculation (three tumors per week). Representative IHF (confocal microscopy) analysis of Gr-1 (MDSCs in green) and TCRγδ (γδT cells in red) on pancreatic tumor frozen sections for each week. The nuclei (in blue) are stained with Draq5. Scale bar 25 μm.

Supplementary Video 1 A direct physical contact between MDSC and Treg interactions in pancreatic tumors. Tumor 1's 3D video from LSM analysis. This video was done with the Imaris software. In green are the blood vessels stained with an anti-alpha SMA (smooth muscle actin), a pericyte marker. The blue and red spots represent, respectively, the Gr-1 and the Foxp3 positive cells. The pink and yellow spots are the Gr-1 and Foxp3 positive cells in interaction (determined with a MatLab plugin associated with Imaris software).

Supplementary Video 2 Assessment of MDSC and Treg interactions in pancreatic tumors by videomicoscopy. Example of film showing persistent interactions.

Supplementary Video 3 Assessment of MDSC and Treg interactions in pancreatic tumors by videomicoscopy. Example of film showing transient interactions.

Supplementary Video 4 One week post-tumor inoculation. 3D video from LSM analysis to determine dynamic interactions between MDSC and Treg cells in pancreatic tumors at 1 week post-tumor inoculation. In green are the blood vessels stained with an anti-alpha SMA. The blue and red spots represent, respectively, the Gr-1 and the Foxp3 positive cells. The yellow spots are the Gr-1 and Foxp3 positive cells in interaction. Three tumors have been analyzed with similar results. Representative 3D video for one of these tumors are shown.

Supplementary Video 5 Two weeks post-tumor inoculation. 3D video from LSM analysis showing dynamic interaction between MDSC and Treg cells in pancreatic tumors at 2 weeks post-tumor inoculation. Three tumors have been analyzed with similar results. Representative 3D video for one of these tumors are shown.

Supplementary Video 6 Three weeks post-tumor inoculation. 3D video from LSM analysis showing dynamic interaction between MDSC and Treg cells in pancreatic tumors at 3 weeks post-tumor inoculation. Three tumors have been analyzed with similar results. Representative 3D video for one of these tumors are shown.

Supplementary Video 7 Four weeks post-tumor inoculation. 3D video from LSM analysis showing dynamic interaction between MDSC and Treg cells in pancreatic tumors at 4 weeks post-tumor inoculation. Three tumors have been analyzed with similar results. Representative 3D video for one of these tumors are shown.

## Abbreviations

PDAC: Pancreatic ductal adenocarcinoma
TME: Tumor microenvironment
TAMs: Tumor-associated macrophages
Tregs: Regulatory T cells
MDSCs: Myeloid-derived suppressor cells
TB mice: Tumor-bearing mice
ROS: Reactive Oxygen Species
RT: Room temperature
FCS: Fetal calf serum.

## References

[1] Rahib L Smith BD Aizenberg R Rosenzweig AB Fleshman JM Matrisian LM. Projecting cancer incidence and deaths to 2030: the unexpected burden of thyroid, liver, and pancreas cancers in the United States. Cancer Res. (2014) 10.1158/0008-5472.CAN-14-0155 24840647

[2] Costello E Greenhalf W Neoptolemos JP. New biomarkers and targets in pancreatic cancer and their application to treatment. Nat Rev Gastroenterol Hepatol. (2012) 10.1038/nrgastro.2012.119 22733351

[3] Neesse A Michl P Frese KK Feig C Cook N Jacobetz MA Stromal biology and therapy in pancreatic cancer. Gut. (2011) 10.1136/gut.2010.226092 20966025

[4] Provenzano PP Cuevas C Chang AE Goel VK Von Hoff DD Hingorani SR. Enzymatic targeting of the stroma ablates physical barriers to treatment of pancreatic ductal adenocarcinoma. Cancer Cell. (2012) 10.1016/j.ccr.2012.01.007 22439937 PMC3371414

[5] Schnurr M Duewell P Bauer C Rothenfusser S Lauber K Endres S. Strategies to relieve immunosuppression in pancreatic cancer. Immunotherapy. (2015) 10.2217/imt.15.9 25917628

[6] Stromnes IM Schmitt TM Hulbert A Brockenbrough JS Nguyen H Cuevas C. T cells engineered against a native antigen can surmount immunologic and physical barriers to treat pancreatic ductal adenocarcinoma. Cancer Cell. (2015) 10.1016/j.ccell.2015.09.022 26525103 PMC4724422

[7] Gabrilovich DI Ostrand-Rosenberg S Bronte V. Coordinated regulation of myeloid cells by tumours. Nat Rev Immunol. (2012) 12:253–68. 10.1038/nri3175 22437938 PMC3587148

[8] Bronte V Brandau S Chen SH Colombo MP Frey AB Greten TF. Recommendations for myeloid-derived suppressor cell nomenclature and characterization standards. Nat Commun. (2016) 7:12150. 10.1038/ncomms12150 27381735 PMC4935811

[9] Ostrand-Rosenberg S Fenselau C. Myeloid-derived suppressor cells: immune-suppressive cells that impair antitumor immunity and are sculpted by their environment. J Immunol. (2018) 200:422–31. 10.4049/jimmunol.1701019 29311384 PMC5765878

[10] Bayne LJ Beatty GL Jhala N Clark CE Rhim AD Stanger BZ. Tumor-derived granulocyte-macrophage colony-stimulating factor regulates myeloid inflammation and T cell immunity in pancreatic cancer. Cancer Cell. (2012) 10.1016/j.ccr.2012.04.025 22698406 PMC3575028

[11] Pylayeva-Gupta Y Lee KE Hajdu CH Miller G Bar-Sagi D. Oncogenic Kras-induced GM-CSF production promotes the development of pancreatic neoplasia. Cancer Cell. (2012) 10.1016/j.ccr.2012.04.024 22698407 PMC3721510

[12] Clark CE Hingorani SR Mick R Combs C Tuveson DA Vonderheide RH. Dynamics of the immune reaction to pancreatic cancer from inception to invasion. Cancer Res. (2007) 10.1158/0008-5472.CAN-07-0175 17909062

[13] Stromnes IM Brockenbrough JS Izeradjene K Carlson MA Cuevas C Simmons RM. Targeted depletion of an MDSC subset unmasks pancreatic ductal adenocarcinoma to adaptive immunity. Gut. (2014) 63:1769–81. 10.1136/gutjnl-2013-306271 24555999 PMC4340484

[14] Mundy-Bosse BL Young GS Bauer T Binkley E Bloomston M Bill MA. Distinct myeloid suppressor cell subsets correlate with plasma IL-6 and IL-10 and reduced interferon-alpha signaling in CD4+ T cells from patients with GI malignancy. Cancer Immunol Immunother. (2011) 10.1007/s00262-011-1029-z 21604071 PMC3521517

[15] Gabitass RF Annels NE Stocken DD Pandha HA Middleton GW. Elevated myeloid-derived suppressor cells in pancreatic, esophageal and gastric cancer are an independent prognostic factor and are associated with significant elevation of the Th2 cytokine interleukin-13. Cancer Immunol Immunother. (2011) 10.1007/s00262-011-1028-0 21644036 PMC3176406

[16] Porembka MR Mitchem JB Belt BA Hsieh CS Lee HM Herndon J. Pancreatic adenocarcinoma induces bone marrow mobilization of myeloid-derived suppressor cells which promote primary tumor growth. Cancer Immunol Immunother. (2012) 10.1007/s00262-011-1178-0 22215137 PMC3697836

[17] Oleinika K Nibbs RJ Graham GJ Fraser AR. Suppression, subversion and escape: the role of regulatory T cells in cancer progression. Clin Exp Immunol. (2013) 10.1111/j.1365-2249.2012.04657.x 23199321 PMC3530093

[18] Nishikawa H Sakaguchi S. Regulatory T cells in cancer immunotherapy. Curr Opin Immunol. (2014) 27:1–7. 10.1016/j.coi.2013.12.005 24413387

[19] Finotello F Trajanoski Z. New strategies for cancer immunotherapy: targeting regulatory T cells. Genome Med. (2017) 10.1186/s13073-017-0402-8 28129791 PMC5273791

[20] Wang X Lang M Zhao T Feng X Zheng C Huang C. Cancer-FOXP3 directly activated CCL5 to recruit FOXP3(+)Treg cells in pancreatic ductal adenocarcinoma. Oncogene. (2017) 36:3048–58. 10.1038/onc.2016.458 27991933 PMC5454319

[21] Tan MC Goedegebuure PS Belt BA Flaherty B Sankpal N Gillanders WE. Disruption of CCR5-dependent homing of regulatory T cells inhibits tumor growth in a murine model of pancreatic cancer. J Immunol. (2009) 10.4049/jimmunol.182.3.1746 19155524 PMC3738070

[22] Wachsmann MB Pop LM Vitetta ES. Pancreatic ductal adenocarcinoma: a review of immunologic aspects. J Investig Med. (2012) 10.2310/JIM.0b013e31824a4d79 22406516 PMC3319488

[23] Fujimura T Ring S Umansky V Mahnke K Enk AH. Regulatory T cells stimulate B7-H1 expression in myeloid-derived suppressor cells in ret melanomas. J Invest Dermatol. (2012) 132:1239–46. 10.1038/jid.2011.416 22189788

[24] Serafini P Mgebroff S Noonan K Borrello I. Myeloid-derived suppressor cells promote cross-tolerance in B-cell lymphoma by expanding regulatory T cells. Cancer Res. (2008) 68:5439–49. 10.1158/0008-5472.CAN-07-6621 18593947 PMC2887390

[25] Schlecker E Stojanovic A Eisen C Quack C Falk CS Umansky V. Tumor-infiltrating monocytic myeloid-derived suppressor cells mediate CCR5-dependent recruitment of regulatory T cells favoring tumor growth. J Immunol. (2012) 10.4049/jimmunol.1201018 23152559

[26] Centuori SM Trad M LaCasse CJ Alizadeh D Larmonier CB Hanke NT. Myeloid-derived suppressor cells from tumor-bearing mice impair TGF-β-induced differentiation of CD4^+^CD25^+^FoxP3^+^ Tregs from CD4^+^CD25-FoxP3- T cells. J Leukoc Biol. (2012) 92:987–97. 10.1189/jlb.0911465 22891289 PMC3476240

[27] Chai MG Kim-Fuchs C Angst E Sloan EK. Bioluminescent orthotopic model of pancreatic cancer progression. J Vis Exp. 50395. 10.3791/50395 23852391 PMC3734868

[28] Anz D Mueller W Golic M Kunz WG Rapp M Koelzer VH. CD103 is a hallmark of tumor-infiltrating regulatory T cells. Int J Cancer. (2011) 10.1002/ijc.25902 21207371

[29] Holmgaard RB Zamarin D Li Y Gasmi B Munn DH Allison JP. Tumor-expressed IDO recruits and activates MDSCs in a Treg-dependent manner. Cell Rep. (2015) 10.1016/j.celrep.2015.08.077 26411680 PMC5013825

[30] Amedei A Niccolai E Prisco D. Pancreatic cancer: role of the immune system in cancer progression and vaccine-based immunotherapy. Hum Vaccin Immunother. (2014) 10.4161/hv.34392 25483688 PMC4514060

[31] Kunk PR Bauer TW Slingluff CL Rahma OE. From bench to bedside a comprehensive review of pancreatic cancer immunotherapy. J Immunother Cancer. (2016) 10.1186/s40425-016-0119-z 26981244 PMC4791889

[32] Pan PY Ma G Weber KJ Ozao-Choy J Wang G Yin B. Immune stimulatory receptor CD40 is required for T-cell suppression and T regulatory cell activation mediated by myeloid-derived suppressor cells in cancer. Cancer Res. (2010) 10.1158/0008-5472.CAN-09-1882 19996287 PMC2805053

[33] Yang R Cai Z Zhang Y Yutzy WH IV Roby KF Roden RB. CD80 in immune suppression by mouse ovarian carcinoma-associated Gr-1^+^CD11b^+^ myeloid cells. Cancer Res. (2006) 66:6807–15. 10.1158/0008-5472.CAN-05-3755 16818658

