# Supplementary figures and images for: Deciphering the Crosstalk Between Myeloid-Derived Suppressor Cells and Regulatory T Cells in Pancreatic Ductal Adenocarcinoma
Source: Front Immunol. 2020 Jan 22;10:3070. doi: 10.3389/fimmu.2019.03070 (PMC6987391; doi:10.3389/fimmu.2019.03070)

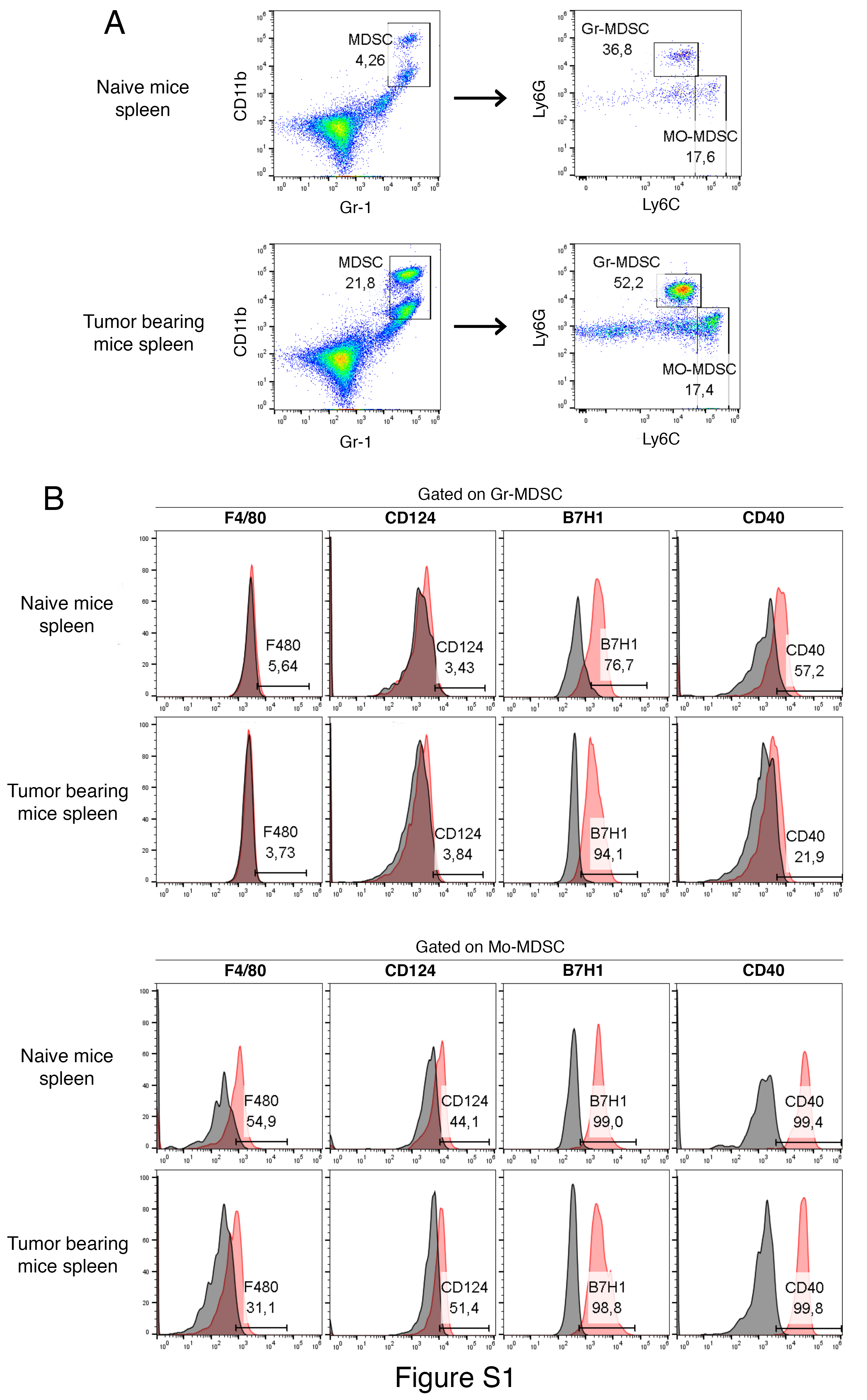

Supplement: Supplementary Figure 1 — Characterization of MDSCs, Treg cells and effector T cells in the spleen of PDAC mice. C57BL/6 mice were injected with Panc02 cells in the pancreas and spleens were harvested 3 weeks post-inoculation. Spleens from naive mice were used as controls. Flow cytometry analysis of (A) MDSC recruitment and (B) of the expression of surface molecules F4/80, CD124, CD40, and B7H1 on Gr-MDSCs (CD11b+Gr-1+Ly6G+Ly6Clow) and Mo-MDSCs (CD11b+Gr-1+Ly6G−Ly6Chigh) was performed. Gray histograms represent isotype control and red histograms specific staining as indicated. Percentage of positive cells are shown. Three independent experiments have been performed with similar results. Representative dot plots and histograms for one of these experiments are shown. [file Image_1.TIF]

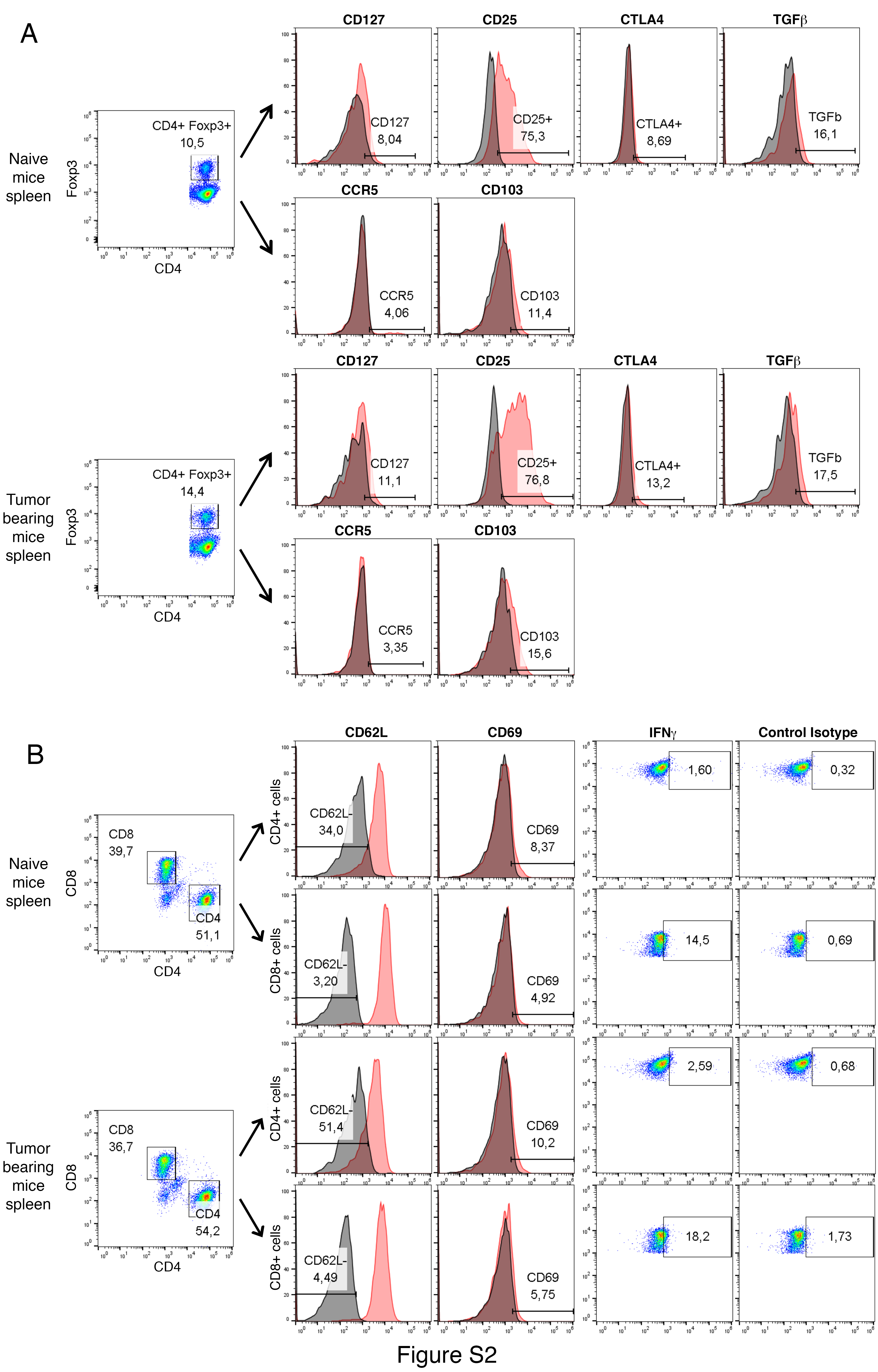

Supplement: Supplementary Figure 2 — Characterization of Treg cells and effector T cells in the spleen of PDAC mice. C57BL/6 mice were injected with Panc02 cells in the pancreas and spleens were harvested 3 weeks post-inoculation. Spleens from naive mice were used as controls. Flow cytometry analysis of (A) the surface molecules CD127, CCR5, CTLA4, TGF-β, and CD103 on Treg (CD45+CD4+ Foxp3+), (B) the activation markers CD62L and CD69, as well as the intracellular expression of IFN-γ on CD4+ and CD8+ T cells. Gray histograms represent isotype control and red histograms specific staining as indicated. Percentage of positive cells are shown. Three independent experiments have been performed. Representative dot plots and histograms for one are shown. [file Image_2.TIF]

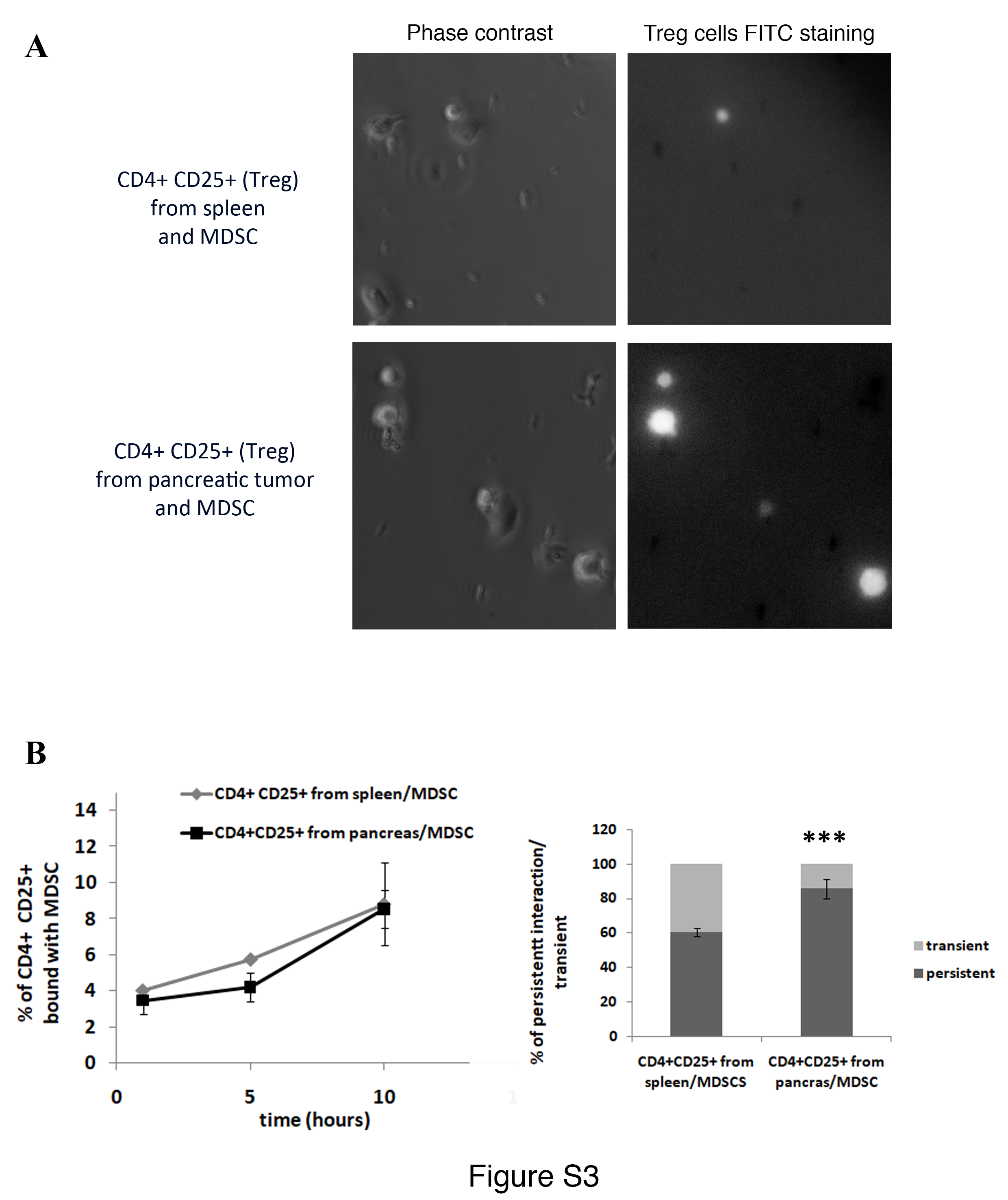

Supplement: Supplementary Figure 3 — Assessment of MDSC and Treg interactions in pancreatic tumors by videomicoscopy. Treg (CD4+CD25+) cells purified both from spleen or pancreas of tumor-bearing mice were loaded with FITC cell tracker and incubated with tumoral MDSCs for 10 h. Microscopic acquisition was performed every 20 min (A) and the percentage of total CD4+CD25+ cells bound to MDSCs was quantified 5 and 10 h post-co-culture. The ratio of persistent interactions (more than 40 min) and transient interactions was also reported (B). ***Mann and Whitney analysis with p value < 0.001%. [file Image_3.TIF]

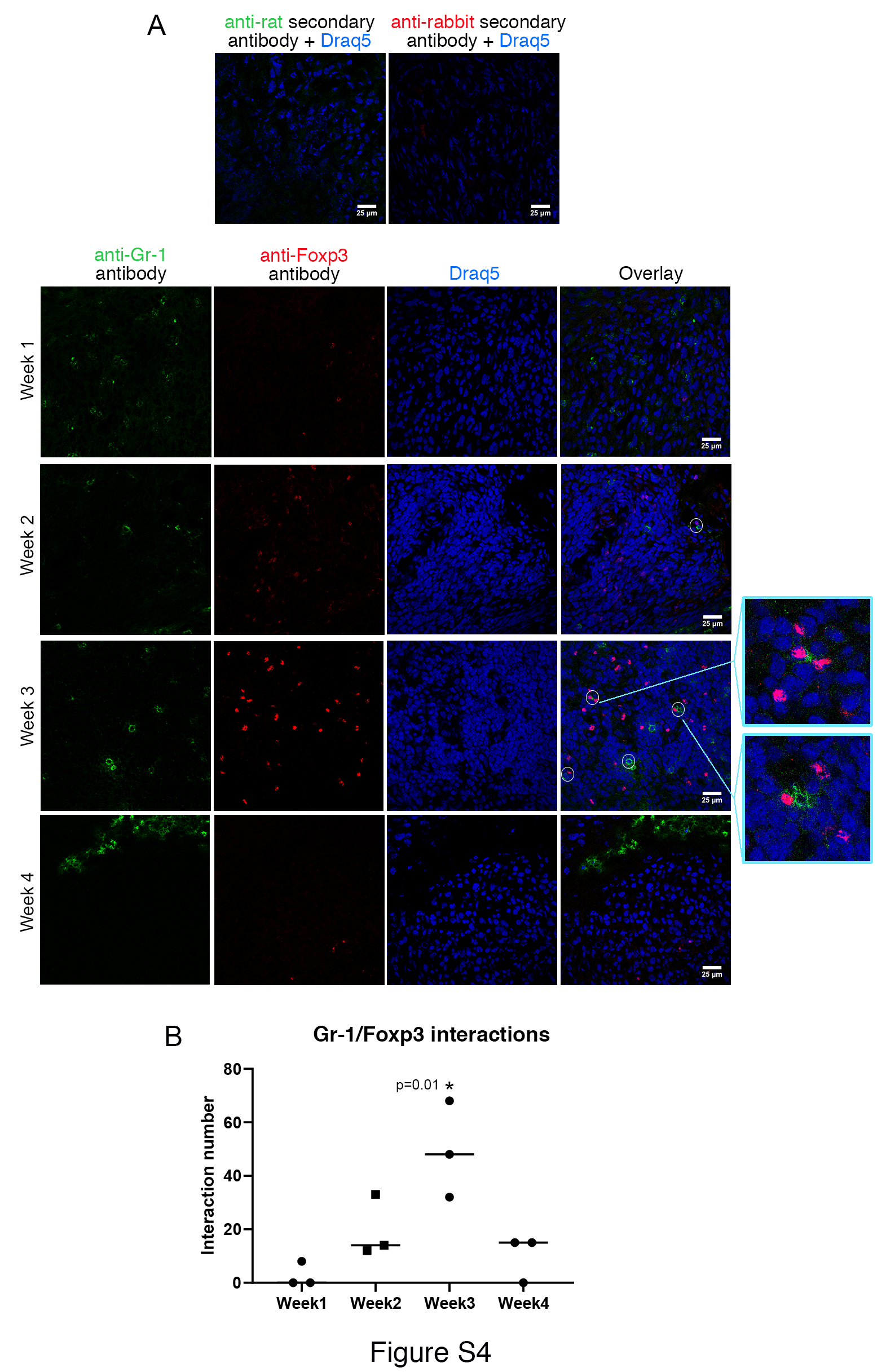

Supplement: Supplementary Figure 4 — Dynamic interactions between MDSCs and Treg cells in orthotopic mouse model of pancreatic cancer. Panc02 cells were injected in the pancreas of C57BL/6 mice and tumors were harvested 1, 2, 3, and 4 weeks post-inoculation (three tumors per week). (A) Representative IHF (confocal microscopy) analysis of Gr-1 (MDSCs in green) and Foxp3 (Treg cells in red) on pancreatic tumor frozen sections for each week. The nuclei (in blue) are stained with Draq5, a fluorescent DNA marker. Zooms show proximity between the different immune cells. Scale bar 25 μm. (B) Light sheet microscopy analysis of Gr-1 (MDSCs) and Foxp3 (Treg cells) on whole cleared pancreatic tumor. Histogram represents quantification of interactions from three whole cleared tumors calculated with a matLab function associated with the Imaris software. See also Supplementary Video 4 (1 week post-tumor inoculation), Supplementary Video 5 (2 weeks post-tumor inoculation), Supplementary Video 6 (3 weeks post-tumor inoculation), and Supplementary Video 7 (4 weeks post-tumor inoculation). [file Image_4.TIF]

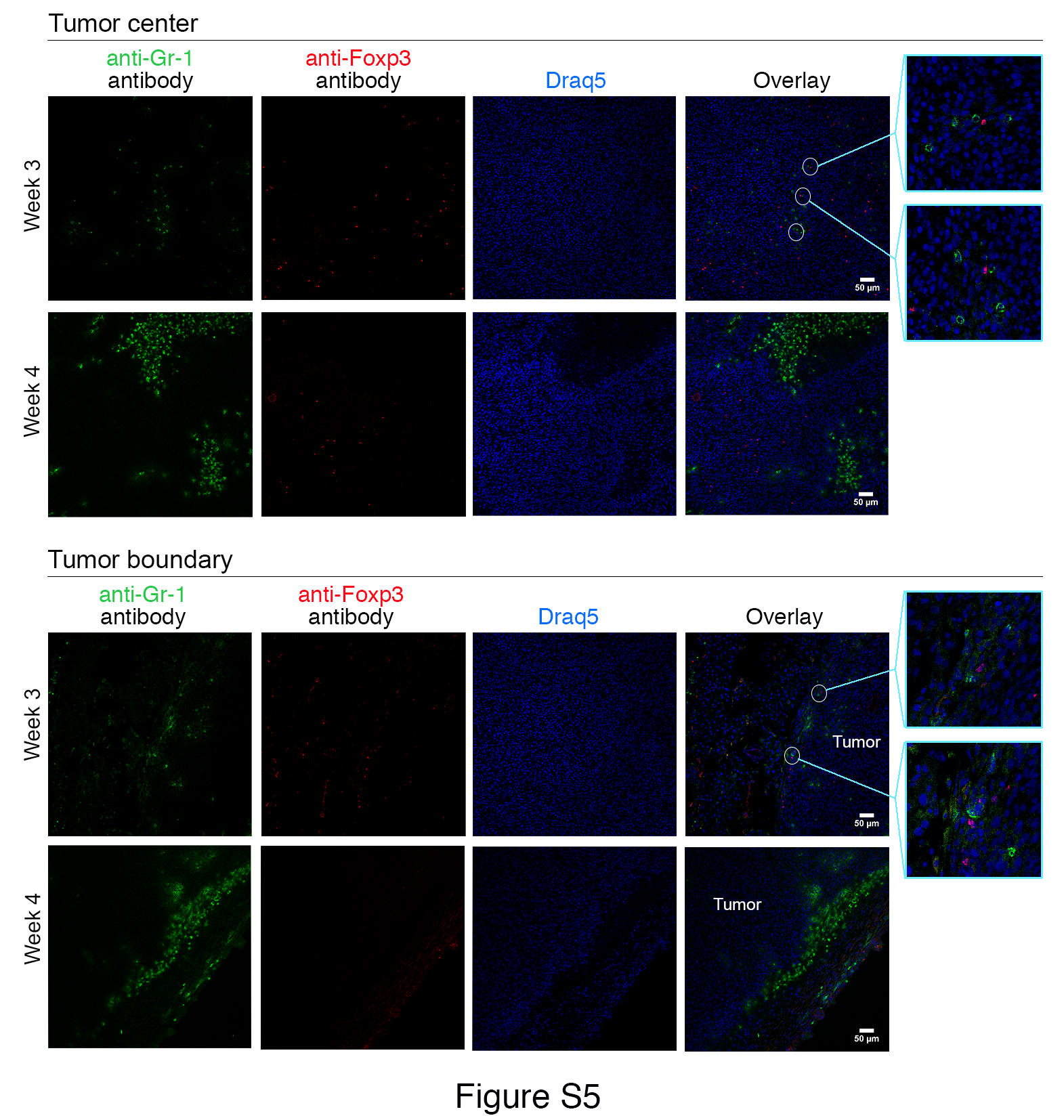

Supplement: Supplementary Figure 5 — Direct interactions between MDSCs and Treg cells in the center and boundary of tumors harvested at 3 and 4 weeks post-inoculation (three tumors per week). Representative IHF (confocal microscopy) analysis of Gr-1 (MDSCs in green) and Foxp3 (Treg cells in red) on pancreatic tumor frozen sections for each week. The nuclei (in blue) are stained with Draq5. Zooms show proximity between the different immune cells. Scale bar 50 μm. See also Supplementary Video 6 (3 weeks post-tumor inoculation) and Supplementary Video 7 (4 weeks post-tumor inoculation). [file Image_5.TIF]

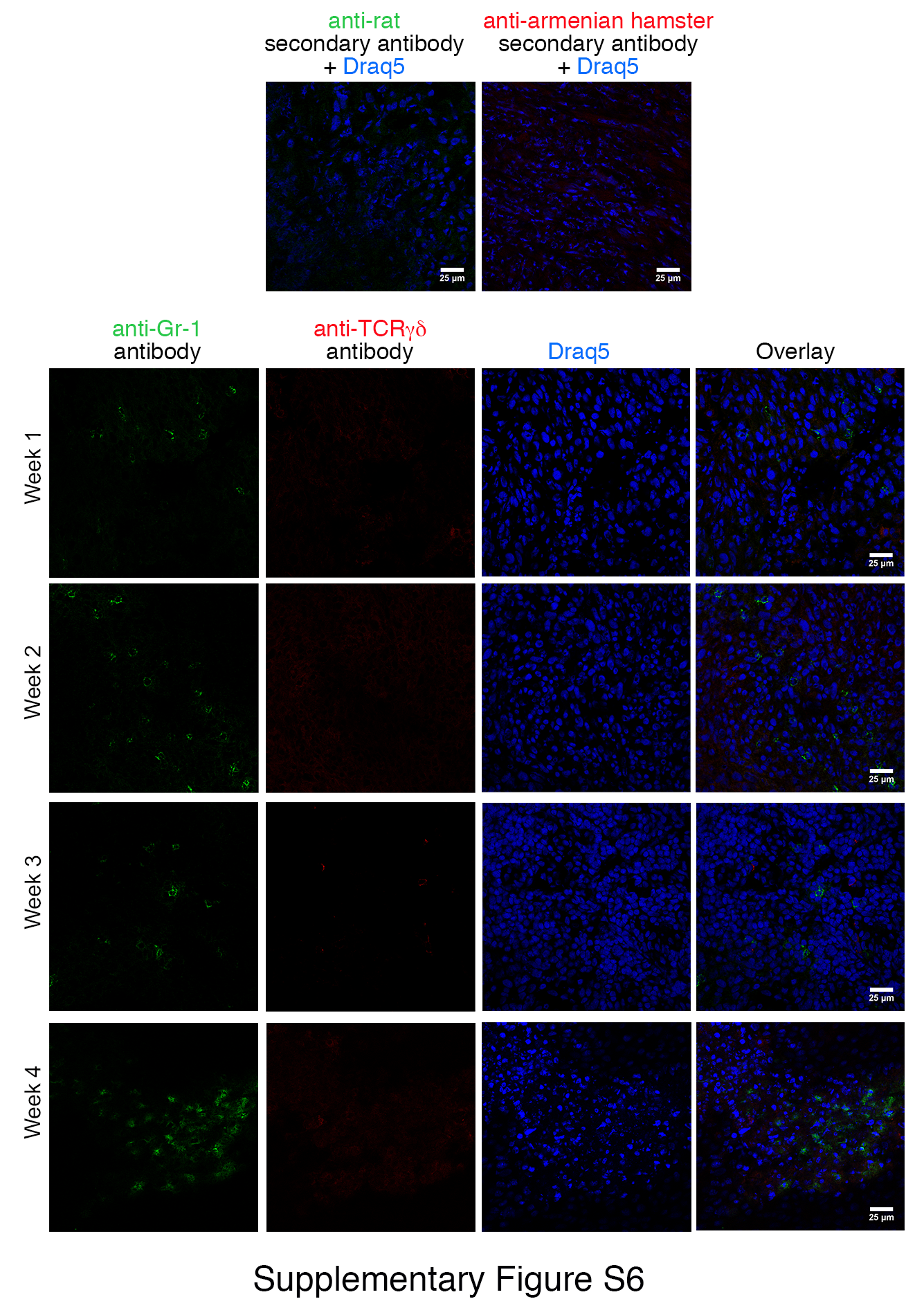

Supplement: Supplementary Figure 6 — Assessment of interactions between MDSCs and γδT cells in orthotopic mouse model of pancreatic cancer. Panc02 cells were injected in the pancreas of C57BL/6 mice and tumors were harvested 1, 2, 3, and 4 weeks post-inoculation (three tumors per week). Representative IHF (confocal microscopy) analysis of Gr-1 (MDSCs in green) and TCRγδ (γδT cells in red) on pancreatic tumor frozen sections for each week. The nuclei (in blue) are stained with Draq5. Scale bar 25 μm. [file Image_6.TIF]
